# Natural Affinity Driven Modification by Silicene to Construct a “Thermal Switch” for Tumorous Bone Loss

**DOI:** 10.1002/advs.202404534

**Published:** 2024-07-21

**Authors:** Yi‐Xing Chen, Yi‐Ping Luo, Xiao‐Dong Hou, Lei Zhang, Tian‐Long Wang, Xi‐Fan Li, Zhi‐Qing Liu, Jin‐Hui Zhao, Aihemaitijiang Aierken, Zhu‐Yun Cai, Bing‐Qiang Lu, Shuo Tan, Xin‐Yu Zhao, Feng Chen, Zi‐Fei Zhou, Long‐Po Zheng

**Affiliations:** ^1^ Department of Orthopedics Shanghai Tenth People's Hospital School of Medicine Tongji University Shanghai 200072 China; ^2^ Department of Orthopedics Second Affiliated Hospital of Naval Medical University 415 Fengyang Road Shanghai 200003 P. R. China; ^3^ Shanghai Key Laboratory of Craniomaxillofacial Development and Diseases Shanghai Stomatological Hospital & School of Stomatology Fudan University Shanghai 201102 P. R. China; ^4^ Shanghai Tenth People's Hospital Chongming Branch Shanghai 202150 China

**Keywords:** integrated bone reconstruction, organic–inorganic hybridization, silicene, thermal switch, tumorous bone defect

## Abstract

Tumorous bone defects present significant challenges for surgical bio‐reconstruction due to the dual pathological conditions of residual tumor presence and extensive bone loss following excision surgery. To address this challenge, a “thermal switch” smart bone scaffold based on the silicene nanosheet‐modified decalcified bone matrix (SNS@DBM) is developed by leveraging the natural affinity between collagen and silicene, which is elucidated by molecular dynamics simulations. Benefitting from its exceptional photothermal ability, biodegradability, and bioactivity, the SNS@DBM “thermal switch” provides an integrated postoperative sequential thermotherapy for tumorous bone loss by exerting three levels of photothermal stimulation (i.e., strong, moderate, and nonstimulation). During the different phases of postoperative bioconstruction, the SNS@DBM scaffold realizes simultaneous residual tumor ablation, tumor recurrence prevention, and bone tissue regeneration. These biological effects are verified in the tumor‐bearing nude mice of patient‐derived tissue xenografts and critical cranium defect rats. Mechanism research prompts moderate heat stimulus generated by and coordinating with SNSs can upregulate osteogenic genes, promote macrophages M2 polarization, and intensify angiogenesis of H‐type vessels. This study introduces a versatile approach to the management of tumorous bone defects.

## Graphical abstract

A multifunctional bone graft is fabricated by affinity‐driven modification of silicene nanosheets. This scaffold can be controlled precisely by NIR II transdermally to give different doses of thermotherapy, which denotes an ideal strategy to deal with the dilemma in postoperative treatment of tumorous large bone defects. Thus, the integrated thermal switch therapeutics combining tumor suppression and bone reconstruction can be realized.

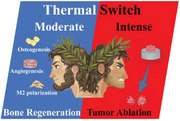

## 1 Introduction

Tumorous bone defect, usually caused by the primary or metastatic malignant tumor in the skeletal system, is a comprehensive disease with coupled pathological conditions of residual tumor presence and extensive bone loss following excision surgery. Bone tumors can lead to huge bone defects through diverse pathways including tumorectomy, tumor invasion, and interference with bone metabolism balance.^[^ ^1^ ^]^ These bone defects need to be repaired after tumor resection to restore the patient's function. Therefore, functional bone grafting scaffolds combining tumor therapy modalities and bone repair components have recently attracted extensive attention. Among different tumor therapy modalities, photothermal therapy (PTT) which employs photothermal agents to convert electromagnetic radiation to heat tumors, is particularly suitable for treating bone tumors due to its advantages in minimal invasiveness, high specificity, and minimal toxicity.^[^ ^2^, ^3^ ^]^ Although various photothermal agents have been introduced to bone scaffolds to generate photothermal effects, many traditional photothermal agents composed of inert materials are unsuitable for bone repair due to their long‐term safety concerns caused by poor biodegradability.^[^ ^4^, ^5^ ^]^ Therefore, degradable photothermal agents, especially those whose degradation products have bone repair‐promoting effects, are still highly required for constructing photothermal bone repair scaffolds.

Single‐element 2D materials (e.g., graphene, black phosphorus, etc.), composed of one or a few layers of one type of atoms, have been used in the field of nanomedicine due to their excellent photothermal properties.^[^ ^6^ ^]^ Their degradation products are usually nontoxic oxygen‐containing acid ions corresponding to the constituent elements, such as carbonate and phosphate ions.^[^ ^7^ ^]^ Among various single‐element 2D materials, silicene has attracted increasing attention due to the excellent biocompatibility, biodegradability, and bioactivity of its degradation products.^[^ ^8^ ^]^ Silicene can produce high‐level hyperthermia to ablate tumors under near‐infrared irradiation followed by degradation to silicate ions that can promote angiogenesis and osteogenesis. Therefore, silicene is an ideal photothermal agent for constructing photothermal bone scaffolds.

2D materials are usually encapsulated by large biomolecules (e.g., proteins, polysaccharides, etc.) when administrated in a biological microenvironment due to their high surface free energy.^[^ ^9^ ^]^ The encapsulation process has been proved to be dynamically driven by the affinity (including covalent and noncovalent) between nanomaterials and large biomolecules.^[^ ^10^ ^]^ It has been demonstrated that noncovalent bindings are the main force of the combination between proteins and 2D nanomaterials,^[^ ^11^ ^]^ including electrostatic interactions, hydrogen bonding, hydrophobic interaction, van der Waals forces, and π–π stacking interactions.^[^ ^12^, ^13^, ^14^ ^]^ These binding modes may cause shifts in some inherent natures of 2D materials such as biodegradation, bioactivity, toxicity, systemic dispersibility, and recyclability of nanomaterials.^[^ ^15^ ^]^ Therefore, the binding force can and should be used to design and fabricate advanced functional scaffolds, but little research has been conducted on it.

**Scheme 1:**
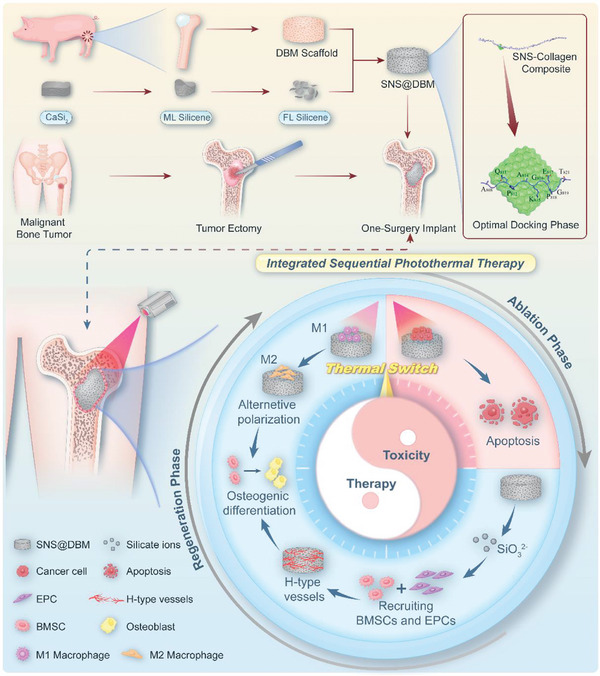
The fabrication and mechanism of the SNS@DBM for treating tumorous bone defects. (ML: multi‐layers, FL: few‐layers).

In this study, we have fabricated a “thermal switch” bone scaffold based on silicene nanosheets modified decalcified bone matrix (SNS@DBM) to provide the integrated postoperative treatment for large tumorous bone loss. The modification is driven by the natural affinity between silicene and collagen type I (COL1). To reveal the binding mechanism, a computer simulation model and the molecular dynamic digital system are constructed. The results of molecular simulation suggest that the SNS can form a relatively good hydrophobic mutual match with surrounding amino acids, which plays a leading role in the combination of SNS and COL1A1 chain. This functional modification endows conventional DBM scaffolds with new characteristics of photothermal conversion and osteogenesis, providing an ideal strategy to address the dilemma in the postoperative treatment of tumorous bone defects. Furthermore, the “thermal switch” bone scaffold can be noninvasively modulated by tuning the parameters of the laser irradiator, delivering three levels of PTT dosages to exert strong, moderate, or nonthermal stimulation during different phases of postoperative treatment. Using our “thermal switch,” tumor ablation and osteogenesis can be realized in one surgery of excision with transplantation (**Scheme** **1**).

## 2 Results and Discussion

### 2.1 Preparation of Silicene Nanosheets

**Figure 1:**
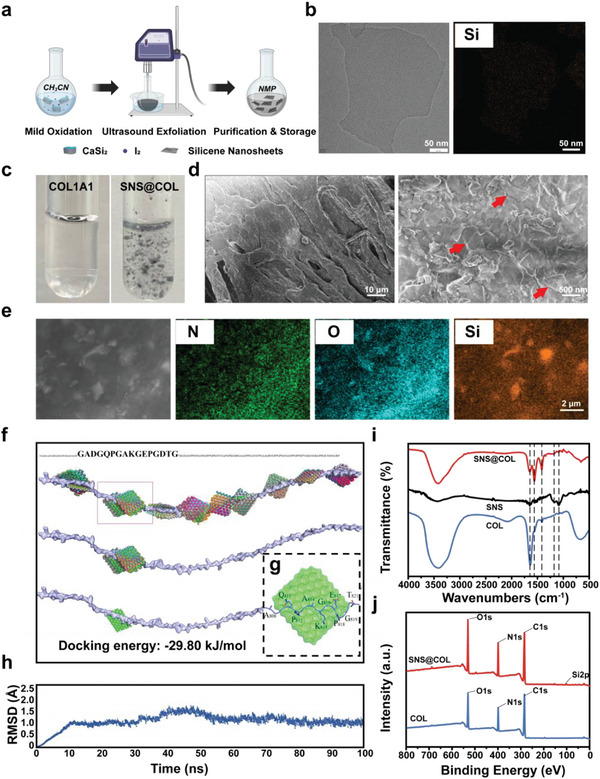
Synthesis of SNSs and affinity to collagen type I. a) Scheme of synthesis of silicene nanosheets (SNSs). b) TEM image of monolayer SNS and its elemental mapping image. c) Photographs of SNS@COL composites (right) and collagen solution (left) at pH 7.4. d) SEM image (left: low‐power view; right: high‐power view; red arrow: SNSs) and e) elemental mapping image of SNS@COL (images share the same scale bar of 2 µm). f) Docking sites and docking phases between SNS and COL1A1 (from up to down: probable docking sites and phases, the optimal docking site, and the docking phase with minimum energy score). g) Detailed interaction phase of the optimal docking phase. h) RMSD value fluctuation of SNS@COL composite. i) Fourier transform infrared (FTIR) spectrum of collagen type I (COL), SNS, and SNS@COL composite. j) XPS survey spectra of collagen (COL) and SNS@COL composite.

The SNSs are prepared by exfoliating silicon using high‐energy ultrasound after a lengthy wet‐chemical mild oxidation. The synthetic strategy to displace the silica and elute the calcium ion by weak oxidizer is analogous to the reported methods for 2D MXene fabrication (**Figure** **1**a).^[^ ^16^, ^17^ ^]^ The lamellar structure of the SNSs is observed by scanning electron microscope (SEM) (Figure S1a, Supporting Information). The details can be observed in the inset image, showing the enlarged view of serried SNSs on an aluminum foil substrate. The component elements including Si, O, and Ca are detected by EDS (Figure S1b, Supporting Information). The existence of oxygen may be due to the oxidation of the ultrathin SNS when exposed to the air.^[^ ^8^ ^]^ The few‐ or single‐layer structure is further confirmed by transmission electron microscope (TEM) images under both bright‐field (Figure 1b) and dark‐field modes (Figure S2a, Supporting Information). The elemental mapping of the TEM image demonstrates that nanosheets almost consist of Si (Figure 1b) with little Ca left (Figure S2b, Supporting Information). The optical absorption spectra of SNSs suspended in deionized water are detected from visible to near‐infrared bands. There exists a broad absorption band with high intensity spanning both the first (NIR I) and the second (NIR II) biowindow (i.e., 1000–1700 nm, Figure S3a, Supporting Information).^[^ ^18^ ^]^ Owing to its better tissue penetration and lower surface thermal effect, the responsiveness of NIR II endows SNSs with a superior bioavailability on PTT.^[^ ^19^ ^]^ The heating curves of SNSs at different concentrations demonstrate their efficient photothermal conversion with the temperature rising to 72 °C under NIR II irradiation (Figure S3b, Supporting Information). All the characterization results certify the successful preparation of single‐ or few‐layer SNSs, and their photothermal property is consistent with that reported in the literature.^[^ ^8^ ^]^

### 2.2 The Exploration of Interaction Mode between SNSs and COL1

2D materials have excellent adsorption capacity due to their high surface energy. Thereby, the as‐prepared SNSs are expected to have high adsorption to biomolecules. Interestingly, the SNSs cause flocculent settlement when added to a clear and transparent collagen aqueous solution (Figure 1c). This phenomenon motivates us to explore the interaction between silene and collagen. The interaction is first characterized by SEM, through which the inlaying of SNSs in collagen fibers can be observed clearly with the element mapping results showing the appearance of Si from SNSs and C, N, and O from collagen (Figure 1d,e). The hydrated size of the SNS‐collagen composite (SNS@COL) is measured by DLS. The size of the SNS@COL is a little larger than that of COL1 (Figure S4a, Supporting Information) and the zeta potential value of COL1 changes from positive to negative after the SNSs modification (Figure S4b, Supporting Information). These results suggest that there exists a natural affinity driving the combination of SNSs and COL1. However, the interaction mode and driving forces remain to be explored further.

To explore the interaction mode between SNS and COL1, a computer molecular model of SNS and COL1 is constructed for computer simulation and molecular dynamics (MD) calculation. The molecular structure of SNS is obtained by ChemDraw and optimized by Chem3D (Figure S5a, Supporting Information). The COL1 has a triple helix structure with two α1 chains and one α2 chain.^[^ ^20^, ^21^ ^]^ By analyzing and comparing the amino acid sequences of collagen type I α1 (COL1A1) chain, there exist abundant repetitive amino acid units of proline–(2S,4R)‐hydroxyproline–glycine (POG), which demonstrates the functional preference of this protein and suggests the single conformability of COL1A1 chain.^[^ ^22^ ^]^

By reason that the realistic structure of COL1 has not been absolutely resolved, the molecular structure of the COL1A1 chain is constructed by homologous reconstruction upon the homologous template of 7JJV from the Research Collaboratory for Structural Bioinformatics Protein Data Bank (RCSB PDB), which is considered reliable whereas their sequence similarity reaches 38.14%.^[^ ^23^ ^]^ In the module MODELLER of program Modeller, the homologous model of COL1A1 is reconstructed under the modeling accuracy of high. The optimal conformation of the COL1A1 molecular model with the size of 354.2 Å and the amino acids number of 127 is obtained using probability density function (PDF) calculation (Figure S5b, Supporting Information).

Then, the long chain of COL1A1 is cut into many fragments and the molecular docking procedure is executed between the SNS and these fragments every 50 Å. This simulation step shows that there are 10 docking sites in the long chain of COL1A1 and each site has 50 docking phases or conformations, totaling 500 phases (Figure 1f). The binding energy of each site is also calculated by the model. The binding energies are all negative under the optimization of molecular dynamics (Table S1, Supporting Information), which signifies that the interaction between SNS and COL1 is spontaneous and their composite is stable. The docking phase ranking is performed according to their binding energy using the cluster analysis module. The results show that all the top 50 docking phases are at the same site on the COL1A1 chain with an amino acid sequence of GADGQPGAKGEPGDTG, indicating that the binding of SNS on the COL1A1 has a fixed and stable site with a strong interaction (Figure 1f).

Subsequently, the binding state is analyzed using MD simulation based on the determined binding site under the parameters of the systemic temperature of 37 °C and the cut‐off distance of 10 Å. The root mean square deviation (RMSD) analysis of the SNS@COL composite is monitored in real time. The RMSD value reaches a plateau after 60 ns simulation, which suggests that there exists a theoretically stable conformation of SNS@COL composite. The plateau starts to form at only 10 ns, implying that the interaction between SNS and COL1A1 is quite strong (Figure 1h). When the simulation system is stable (i.e., RMSD at plateau region), the average structure of the SNS@COL composite is abstracted for interaction mode analysis. As the optimal docking site is confirmed and the average combination phase is obtained, the detailed interaction mode is further investigated and the bonding energy is calculated. The binding energy of the optimal docking phase is determined to be −29.80 kJ mol^−1^, and the negative value certifies the spontaneous combination of SNS and collagen (Table S1, Supporting Information). To further focus on the optimal docking phase, the flat plains of amino acids at the binding site to adapt SNS can be observed (Figure 1g). Such a flat structure is attributed to the hydrophobic interaction formed between SNS and the amino acids W881, F874, V694, and V702, and the polar hydrogen bonds formed between amino acid L844 and amino acid K877 and S841.

To confirm the molecule junction and possible mechanism from MD simulation, FTIR and XPS characterization are conducted. The XPS survey spectra of collagen and SNS@COL composite confirm the doping of Si in SNS@COL composite (Figure 1j). In the high‐resolution spectrum of Si, the disappearance of Si─Si and the reduction of Si─OH suggests the chemical state of Si has changed (Figure S6a,b, Supporting Information). The increased content of Si─O may be due to the condensation reaction between Si─OH and ─NH_2_. In the FTIR spectra, the changes in transmittance peaks also confirm the linkage of SNS and amino acids from COL1. The typical peeks of C═O at 1760–1595 cm^−1^ and N─H at 3400–2000 cm^−1^ demonstrate the existence of collagen while the typical peeks of Si─O at 1100–1000 cm^−1^ confirm the combination of SNS with COL1 (Figure 1i).

### 2.3 The Fabrication of the “Thermal Switch” SNS@DBM Scaffold

**Figure 2:**
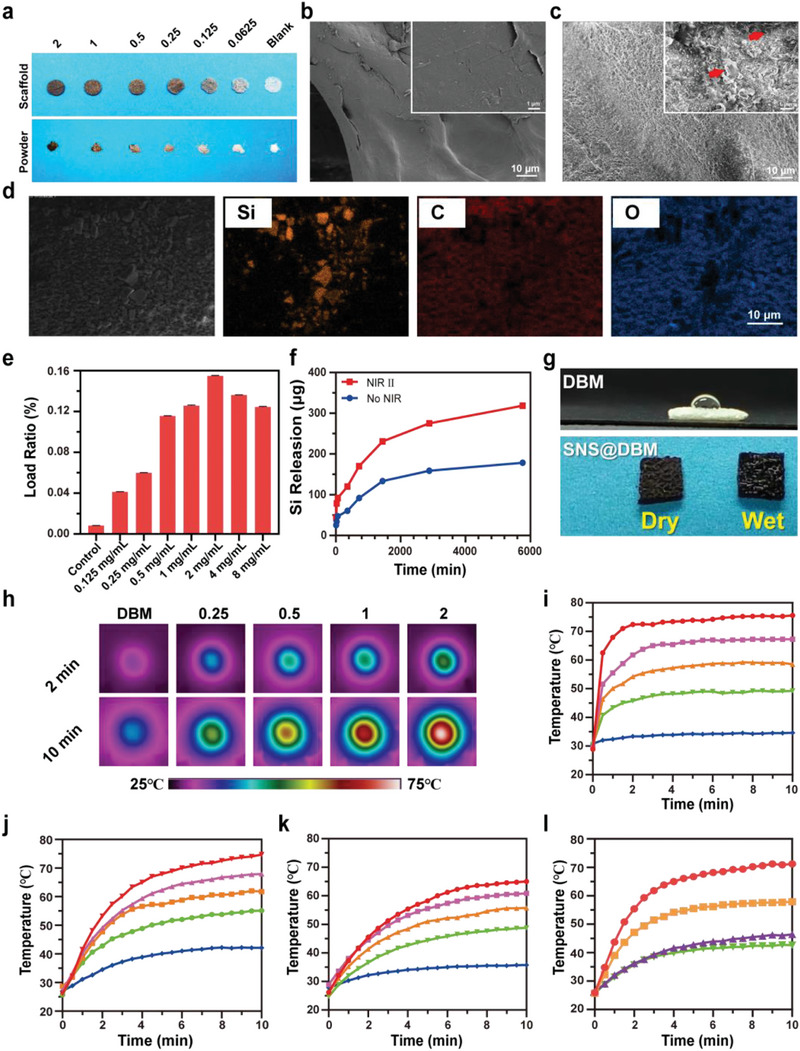
Preparation of SNS@DBM modified scaffold and its properties. a) Digital photos of SNS@DBM scaffolds modified by various concentrations of SNS suspension (from left to right 2, 1, 0.5, 0.25, 0.125, and 0.0625 mg mL^−1^ and Blank). SEM images of b) DBM scaffold and c) SNS@DBM scaffold [inset images in (b) and (c) are high‐power views sharing the bar of 1 µm]. d) Elemental mapping image of SNS@DBM scaffold (images share the same scale bar of 10 µm). e) Loading ratio of Si in SNS@DBM modified by various concentrations of SNS suspension and f) Si release behavior at 37 °C with or without NIR II irradiation. g) Digital photos of comparison between the DBM scaffold and SNS@DBM scaffold with their hydrophobicity. h) Infrared thermal images of SNS@DBM scaffolds modified by various concentrations of SNS suspension (from left to right 0, 0.25, 0.5, 1, and 2 mg mL^−1^) in PBS, and corresponding photothermal‐conversion heating curves (10 min) in i) dry condition (scaffold) and j) wet condition (scaffold). k) Heating curves (10 min) of powder state of SNS@DBM showing varied photothermal‐conversion temperatures in PBS. The SNS@DBM scaffolds were modified by different concentrations of SNS suspension in (i)–(k) (from up to down 2, 1, 0.5, 0.25, and 0 mg mL^−1^). l) Heating curves (10 min) of SNS@DBM modified by 2 mg mL^−1^ SNSs suspension under different output power NIR II irradiation in PBS (from up to down 1.5, 1, 0.75, and 0.5 W; spot size 0.785 cm^2^).

By virtue of the strong interaction between SNS and COL1, the SNS are further used to modify the wildly applied DBM scaffold whose main component is collagen type I. To fit the rat critical skull‐defect model, DBM scaffolds are shaped into homogenous round slices with a diameter of 5 mm and thickness of around 0.8‐1 mm (Figure S7, Supporting Information). The SNS@DBM scaffold is prepared by immersing the DBM scaffold in the SNS suspension. As the concentration of SNS suspension increases, the color of the SNS@DBM scaffold becomes increasingly black (**Figure** **2**a). The morphology characteristics show that the smooth surface of DBM becomes rough after SNS modification (Figure 2b,c). The elemental mapping demonstrates the existence of Si, which also verifies the successful preparation of the SNS@DBM (Figure 2d).

To explore the content of silicon in the SNS@DBM scaffold, the scaffold is dissolved for ICP measurements. The loading of SNS rises from 0.04% to 0.15% (m/m) with the elevating concentration of SNS reaction suspension from 0.125 to 2 mg mL^−1^ (Figure 2e). However, the loading decreases when the concentration is further increased to 4 and 8 mg mL^−1^, which may be attributed to the rouleaux‐like structure formed at the high concentrations to inhibit further adsorption of SNS. Therefore, 2 mg mL^−1^ is used as the optimum concentration of SNS suspension for the modification of the DBM scaffold. The long‐term release property is also evaluated by ICP‐OES. A sustained release curve without any burst release is observed for Si release from the SNS@DBM scaffold (Figure 2f). The release level can be elevated by the irradiation of NIR II over the entire releasing process.

The DBM usually has poor hydrophilicity due to its fabrication processing, which is not conducive to application in the biological environment.^[^ ^24^ ^]^ Our SNS modification strategy improves the hydrophilicity of the DBM scaffold due to the high hydrophilicity of SNS. The water contact experiments show that an aqueous droplet is maintained and held upon the porous surface of DBM, while the droplet infiltrates into the scaffold immediately once attaches to the SNS@DBM (Figure 2g). This process can be observed more clearly in the video (Videos S1 and S2, Supporting Information). Quantitative data is displayed by the measurement of contact angle. The water drop can be held on the surface of the DBM scaffold, while the droplet quickly infiltrates the SNS scaffold (Figure S8a,b, Supporting Information). Thus, the contact angle was detected in a stable state on the DBM scaffold, while it was detected once the water droplet touched the SNS@DBM scaffold. The contact angle is 80.24° on average in the DBM group and 29.04° on average in the SNS@DBM group (Figure S8c, Supporting Information). The result of the contact angle test solid confirms that the modification by SNS can significantly improve the hydrophilicity of the DBM scaffold, which is beneficial for improving biocompatibility and bioavailability.

SNS modification also gifts the DBM scaffold an exceptional photothermal conversion property. To detect the conversion performance, SNS@DBM scaffolds are irradiated by a 1064 nm laser with a collimator (spot size 0.785 cm^2^) under different output powers. The photothermal conversion heating curves are constantly recorded using an infrared camera with the non‐modified DBM scaffold as a control. After 10 min irradiation by NIR II, scaffolds in different groups show different temperature plateaus displayed in infrared heating maps (Figure 2h). In dry conditions, the SNS@DBM scaffold modified with 2 mg mL^−1^ SNSs suspension can reach 75 °C rapidly within 2 min, while the DBM scaffold can only be heated between 30 and 35 °C which is close to the environment temperature (Figure 2i). The peak temperature of SNS@DBM scaffold immersed in PBS has no notable change but the duration to reach the plateau is notably prolonged due to the specific high heat capacity of water (Figure 2j). Compared to the plate‐shaped scaffold, both the maximum heating temperature and heating rate of powders are much lower due to the high specific surface area to dissipate heat to water (Figure 2k). Then the laser output power is modulated from 0.5 to 1.5 W for SNSs modified scaffolds at the SNS concentration of 2 mg mL^−1^. The scaffold modified by 2 mg mL^−1^ SNSs can rise to ≈75 °C under the irradiation of 1.5 W NIR II while it can also maintain at a mild temperature of around 40 °C under the irradiation of 0.5 W NIR II (Figure 2l), which is considered as a suitable temperature for osteogenesis.^[^ ^25^ ^]^ The on/off cycle is plotted to describe the heating and cooling phases (Figure S9a, Supporting Information). Almost identical heating‐cooling cycles are obtained in the five cycled experiments, demonstrating the high photothermal stability of the SNS@DBM scaffold (Figure S9b, Supporting Information).

### 2.4 In Vivo Tumor Ablation by SNS@DBM Scaffold

**Figure 3:**
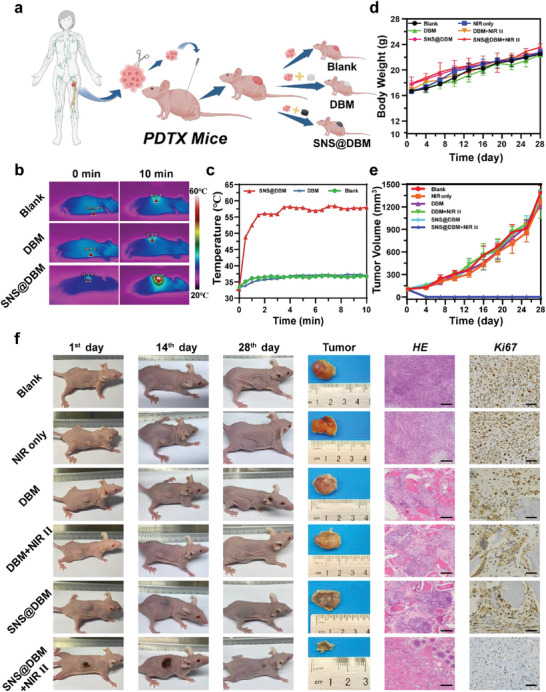
In vivo tumor ablation by PTT. a) Schematic diagram of the establishment of the PDTX model. b) Infrared thermal images of xenografting tumor of different groups (Blank, DBM, and SNS@DBM), and c) corresponding photothermal‐conversion heating curves (10 min). d) Time‐dependent body weight curves of nude mice (*n* = 6, mean ± SD) after different treatments. The treatments were performed only once. e) In vivo PDTX tumor proliferation curves of nude mice (*n* = 6, mean ± SD) after various treatments. f) Digital photographs of tumor‐bearing mice and their tumor sites in 28 d after treatments. The harvest tumors and H&E staining and antigen Ki‐67 staining for cellular proliferation in tumor sections from each group. Images of H&E staining share the same scale bar of 200 µm. Images of Ki‐67 staining share the same scale bar of 50 µm.

The PTT effect of the SNS@DBM scaffold is evaluated through the subcutaneous tumor formation mode of patient‐derived tissue xenografts (PDTX) on Balb/c nude mice. In realistic circumstances, the bone‐filling materials are surrounded by post‐operational residual tumors. After tumor resection operation, residual lesions that invade to surrounding medullary cavity or other soft tissue like muscles are difficult to detect and eliminate.^[^ ^26^ ^]^ When implants are fixed at the post‐operative defect, they are wrapped by residual tumors. To simulate this adjacent relationship, a tumor‐bearing model is established by mixing the patient‐derived tumor tissues and SNS@DBM scaffold powder (**Figure** **3**a).

About one week after implanting the mixture of tumor and scaffold, once the tumor volume reaches around 100–150 mm^3^, a 1064 nm laser is exerted on experimental groups. The temperature is recorded by an infrared camera. When exposed to a 1064 nm laser irradiation, the tumor site containing SNS@DBM rises to a therapeutic temperature of around 58 °C rapidly, which can ablate the solid tumor surrounding the implants. In the Blank and DBM group, there is a little fluctuation around the initial temperature of the body surface (Figure 3b,c). The body weights and tumor volumes are recorded every two days after intervention. The observation lasts for four weeks. Tumors and organs are harvested under euthanasia. The growth of body weight has no distinction in each group after invention, which suggests that the SNS@DBM scaffold and subsequent PTT have negligible impacts on the growth of mice (Figure 3d).

After irradiation, no obvious tumors can be observed in the experimental group (i.e., SNS@DBM + NIR, Figure 3e). Compared to the Blank group, xenograft tumors grown with the DBM scaffolds are brighter and those growing with SNS@DBM scaffolds are darker whether in vivo or being isolated (Figure 3f). This visible change demonstrates the close symbiosis of tumor and scaffold, which simulates the adjacent relationship between residual tumors and bone grafts after surgery. The microscopy images of H&E staining and Ki‐67 immunohistochemistry (IHC) staining show that the osteosarcoma cells in the SNS@DBM + NIR group are eliminated, leaving the collagen fiber of the scar on the tissue slice. In contrast, neither the scaffold or NIR can damage cancer cells alone (Figure 3f). The harvest tumors in different groups also demonstrate that the scaffold or NIR cannot hinder the development of osteosarcoma xenografts, which suggests the tumor ablation is merely caused by the PTT of the SNS@DBM scaffold (Figure S10, Supporting Information). The tumor cell viability experiments also verify the PTT efficacy of the SNS@DBM scaffold under NIR irradiation (Figure S11a,b, Supporting Information).

The damage of the surrounding tissues is always one of the major side effects of PTT. Through the digital photo of treated mice, skin damage and black scars expand during the first period and gradually recover until almost normal in the last duration (Figure 3f). Through H&E staining of the skin lesion, the stratum corneum is severely damaged with massive infiltration of inflammatory cells and the formation of lymphoid follicles in the first week after PTT. The inflammatory response subsides during the next week but the neonatal subcutaneous collagen fibers are disordered and the scar remains on the epidermis. With the repair of burned skin, the subcutaneous collagen tissue is restored to normal, and the epidermal stratum corneum is remolded (Figure S12, Supporting Information). This phenomenon suggests that the wound caused by PTT of the SNS@DBM scaffold is limited and reversible. The secondary enlargement of skin lesions may be caused by inflammatory immunoreaction.

Therefore, in this research, the application of SNSs can effectively balance the advantages and disadvantages of PTT. SNSs possess a splendid responsibility and efficient photothermal conversion ability in the second biowindow of NIR, which makes the precise control of energy release and intervention range realized. Furthermore, with the degradation of SNSs, the release of silicate ions is also favorable for the recovery of soft tissues impaired by PTT.^[^ ^27^ ^]^ The organs harvested from different groups have no difference in histology, which suggests the high bio‐safety of the SNS@DBM scaffold with PTT for in vivo application (Figure S13, Supporting Information).

### 2.5 In Vivo Osteogenic Effect of SNS@DBM Scaffold and Moderate Thermal Stimulation

The reconstruction is a vital proposition after tumor resection. These bone defects caused by bone tumors need to be repaired to restore the patient's function. Therefore, the abilities of residue ablation and bone regeneration are both indispensable for bone grafting materials for tumor treatment. Conventional bone grafts can usually offer mechanical properties only. Some bone tissue engineering (BTE) scaffolds have positive effects on osteogenesis by adding cells, factors, elements, or modification by other bioactive ingredients.^[^ ^28^, ^29^ ^]^

**Figure 4:**
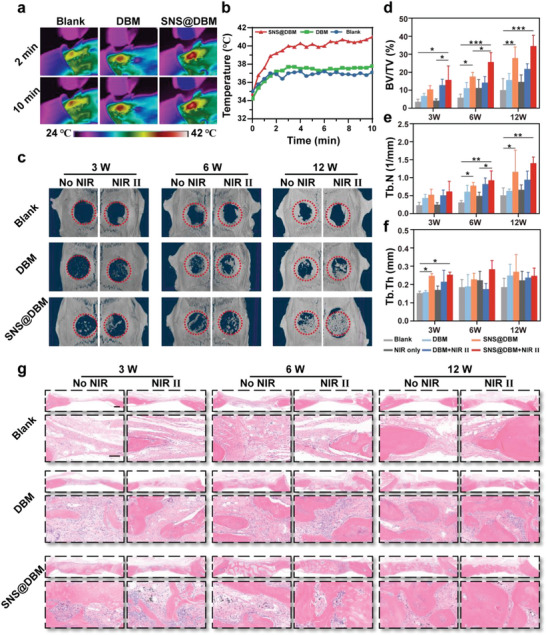
In vivo osteogenic activity of SNS@DBM scaffold and mild PTT. a) Infrared thermal images of defect region of different groups (Blank, DBM, and SNS@DBM), and b) corresponding photothermal‐conversion heating curves (10 min). c) Micro‐CT images of the calvarium defect in rat model at 3, 6, and 12 weeks after implantation of scaffolds, and d–f) the statistical analysis of the bone quality index BV/TV (%), Tb. N (1 mm^−1^), and Tb.Th (mm), respectively (*n* = 4, mean ± SD). g) Hematoxylin and eosin (H&E) staining demonstrating the formation of new bone after implantation of scaffolds in calvarium defects (upper panels present low‐power views sharing the bar of 500 µm; lower panels present high‐power views sharing the bar of 100 µm).

In this study, a multifunctional SNS@DBM scaffold is fabricated as a “thermal switch” to exert different levels of thermal stimuli. After abating residual tumors under intense PTT, a moderate thermal stimulation is delivered to motivate bone regeneration^[^ ^30^ ^]^ followed by offering the bioactive degradation product after the thermal stimulation is off. Critical cranial bone defects of 5 mm are made on the two sides of the SD rat. The DBM and SNS@DBM scaffolds are implanted into the lesion, and the lesion without filling is a blank control (Figure S14, Supporting Information). The in vivo photothermal conversion efficiency of SNS@DBM scaffolds is superior to DBM and blank group under a low‐power NIR of 1 W (**Figure** **4**a). The local temperature in the SNS@DBM group can reach and maintain at 40 ± 1 °C, which is considered a desirable hyperthermal condition for cell metabolism and tissue repair.^[^ ^25^, ^31^ ^]^ While the temperatures in the other two groups only have negligible elevation (Figure 4b).

The irradiation is exerted every five days during the 12‐week experimental period. Corresponding biological samples are harvested for various characterizations. Serum biochemical indicators of liver and renal function have no significant difference in each group (Figure S15a–d, Supporting Information), which demonstrates that the SNS@DBM scaffold with long‐term NIR irradiation is safe for experimental animals.

The reconstructed images of micro‐CT scanning and their statistical data demonstrate that the SNS@DBM scaffold can expedite endogenous bone regeneration in each period of bone repair, which is further reinforced by moderate thermal stimulation (Figure 4c–f). H&E and Masson staining also confirm abundant neo‐genetic bone inside the SNS@DBM scaffold (**Figures** 4g and **5**a). The hierarchical pore structure of the SNS@DBM scaffold is beneficial for the crawling of various functional cells around the lesion to “eat” the implants and regenerate new bone. With the degradation of the SNSs, the degradation product (i.e., silicate) promotes the deposition of calcium, which facilitates the transition of neo‐genetic bone (blue‐stained matrix) to mature bone (red‐stained matrix, Figure 5a).

Figure 5In vivo osteogenesis mechanism of SNS@DBM scaffold and mild PTT. a) Masson's trichrome (MT) staining of the cranial defect (NB: new bone, MB: mature bone. Upper panels present low‐power views sharing the bar of 500 µm; Lower panels present high‐power views sharing the bar of 100 µm). b) Immunohistochemistry staining of Runx2^+^ cells among the cranial defect region (Red stars: Runx2^+^ areas. Images share the same scale bar of 200 µm.) Immunofluorescence staining of c) H‐type vessels (red: CD31^+^, green: EMCN^+^, yellow: Merge. Samples were harvested after six weeks. Left images share the same scale bar of 50 µm. Merge images share the same scale bar of 50 µm), and d) macrophage polarization (Upper panels present M1 cells staining, green: CD68^+^, red: iNOS^+^, orange: Merge. Lower panels present M2 cells staining, green: CD68^+^, pink: CD163^+^, white: Merge. Images share the same scale bar of 50 µm) among the bone regeneration region.

**Figure:**
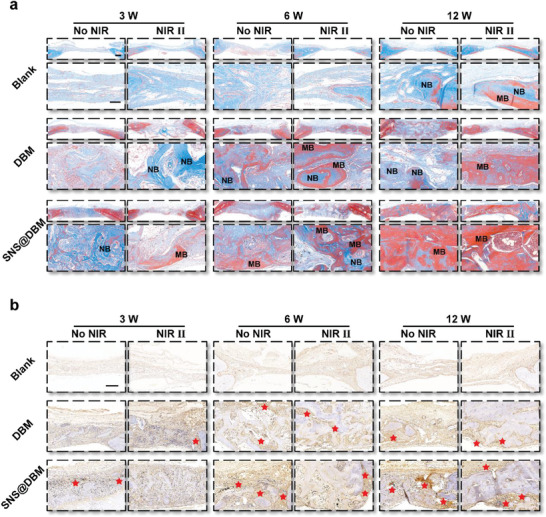

**Figure:**
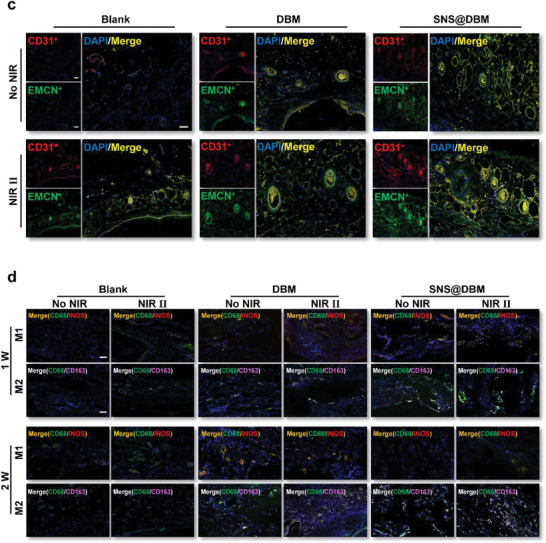

### 2.6 In Vivo Osteogenic Mechanism of SNS@DBM Scaffold and Moderate Thermal Stimulation

The SNS@DBM scaffold coupled with thermal stimulation provides multiple osteogenic mechanisms. On the one hand, the negatively charged SNSs can absorb positively charged calcium ions, and its oxidation product (i.e., Si─OH) can further coordinate with the Ca^2+^, which can initiate and promote the biomineralization of the DBM collagen matrix.^[^ ^32^, ^33^ ^]^ On the other hand, silicene can be easily degraded to silicate ions, which have various bioactivities.^[^ ^34^ ^]^ For example, silicate can promote the formation of blood vessels by activating angiogenic genes like VEGF, PDGF‐BB, and NOS3,^[^ ^35^, ^36^ ^]^ and cartilage regeneration through participating in collagen synthesis.^[^ ^37^, ^38^ ^]^ Besides, silicon can also enhance bone repair by assisting mineral deposit and modulating bone cell metabolism.^[^ ^39^, ^40^, ^41^ ^]^ The ability of our SNS@DBM scaffold to recruit BMSCs and induce osteogenic differentiation is verified through immunohistochemistry staining of Runx2^+^ cells around regeneration areas (Figure 5b).

In addition, the SNS@DBM scaffold possesses the effect of angiogenesis and immunomodulation around the bone grafting implants. Moderate thermal stimulation plays a synergistic role in creating a favorable environment for osteogenesis with the SNS@DBM scaffold.^[^ ^42^, ^43^, ^44^ ^]^ Histological vessels (i.e., H‐type blood vessels) are highly associated with osteogenesis, which are defined by high expression of CD31 and Endomucin (CD31^hi^Emcn^hi^).^[^ ^45^, ^46^ ^]^ Immunofluorescence staining results manifest that the SNS@DBM scaffolds own a considerable biological activity to recruit the endothelial cells to form H‐type blood vessels, which is enhanced by the NIR II irradiation (Figure 5c). The angiogenesis effect initiated by SNSs is confirmed by tube formation assay in vitro directly (Figure S18a,b, Supporting Information). Coupled with the H‐type vessel formation, various kinds of cells and nutrients including Runx2^+^ osteogenic progenitor cells are delivered into the osteogenic microenvironment. This result is consistent with the immunohistochemistry staining of Runx2^+^ cells (Figure 5b).

The inflammatory reaction is quickly activated in the body to deal with the acute injury or exogenous implants, removing foreign objects and gathering gradients for regeneration.^[^ ^47^ ^]^ Macrophage is one of the most regular members of inflammatory cells after injury.^[^ ^48^ ^]^ The naïve macrophage will be activated to the classic phenotype (M1) in the inflammation reaction phase, which is known as a pro‐inflammation immune cell.^[^ ^49^ ^]^ As the process proceeds, the M1 macrophage will be alternatively activated to the anti‐inflammation phenotype M2 which is considered a pro‐repairment agent.^[^ ^50^ ^]^ However, the controlled inflammatory response in the early period is beneficial to the initiation of angiogenesis and the preparation of cells and substances.^[^ ^51^, ^52^, ^53^ ^]^ Thus, “immune‐interactive” materials that can modulate inflammatory response have been considered suitable for implant integration and tissue regeneration.^[^ ^54^ ^]^ Some studies have preliminarily revealed that the reduced silicene nanosheet may possess the potential for anti‐inflammation and promoting M2 polarization for bone repair.^[^ ^55^, ^56^ ^]^ It is also reported that mild photothermal therapy can promote M2 phenotype polarization of macrophages.^[^ ^57^ ^]^ The immunofluorescence staining of iNOS and CD163 results show that the number of M1 macrophages is at the same level in the DBM and SNS@DBM group while the number of M2 macrophages is much increased in the SNS@DBM group in the early period after injury and implantation (Figure 5d). Moderate thermal stimulation can promote M2 polarization in each subgroup. In the middle and late stages, the inflammation phase has shifted to tissue regeneration processes at the injury area. The number of M1 macrophages decreases and the number of M2 macrophages increases in SNS@DBM groups with and without NIR II (Figure 5d). In the DBM group, the proinflammatory M1 phenotype is always at a high level. The application of NIR II irradiation impairs the M1 polarization and promotes the M2 polarization.

### 2.7 Cellular and Molecular Mechanisms for Bone Regeneration of SNS@DBM Scaffold and Moderate Thermal Stimulation

**Figure 6:**
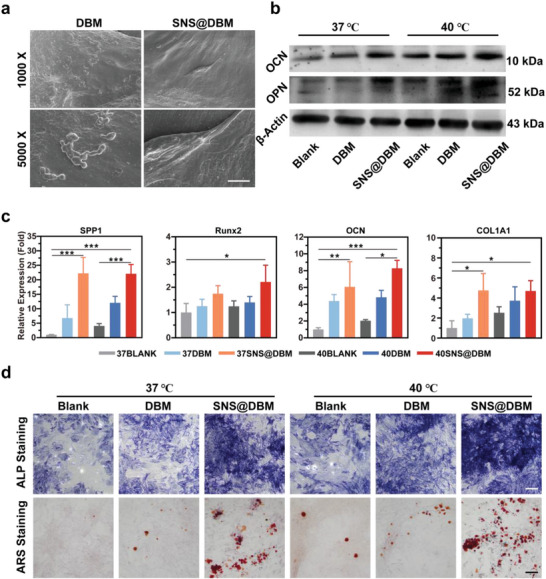
In vitro osteoinductive property and mechanism of SNS@DBM scaffold and mild PTT. a) SEM images of BMSCs on the DBM and SNS@DBM (Upper panels present low‐power views sharing the bar of 25 µm; Lower panels present high‐power views sharing the bar of 5 µm). b) Relative protein expression levels of osteogenic markers OCN and OPN were analyzed by western blot. c) Relative expression of osteogenic genes OCN, SPP1, Runx2, and COLA1 were analyzed by RT‐qPCR (*n* = 4, mean ± SD). d) ALP and alizarin red staining of BMSCs cultured in various treatment groups. Images of ALP staining share the same scale bar of 400 µm. Images of alizarin red staining share the same scale bar of 100 µm.

**Figure 7:**
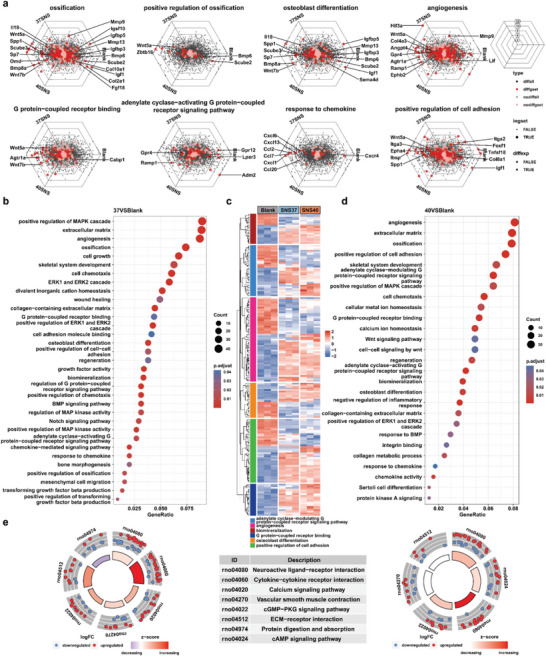
Gene expression profiling of BMSCs between Blank, 37SNS, and 40SNS groups and functional enrichment analysis. a) Distribution of genes for biological processes associated with osteogenesis among overall genes (orientation of dots in hexagonal labels represents upregulation among clusters. logFC > 1, *p* < 0.01 filtered differential genes are highlighted with red dots). Gene ontology enrichment analysis of differential genes: b) comparison between 37SNS and Blank groups, c) heatmap of osteogenesis‐related biological processes differential gene expression in each group, and d) comparison between 40SNS and Blank groups). e) KEGG pathway enrichment analysis of differential genes between groups (left, comparison between 37SNS and Blank groups. right, comparison between 40SNS and Blank groups).

The osteogenesis mechanisms of the SNS@DBM scaffold are explored in vitro. The modification by SNSs not only changes some physical and chemical properties of the DBM scaffold but also brings some physiological changes. The degradation products SiO_3_^2−^ first recruits the BMSCs from the bone marrow around the lesion area. The chemotaxis of BMSCs to Si is affirmed by the transwell assay (Figure S19a,b, Supporting Information). GO analysis of whole transcriptome resequencing shows that SNSs can enhance the response of BMSCs to cell chemokine (Figure 7a,b,d). Due to the improvement of hydrophilicity of the scaffold, when BMSCs gather around the bone grafts, it becomes easier for them to attach to the SNS@DBM scaffold and proliferate on this substrate. The flat morphology of BMSCs and increased pseudopodia can be observed on the scaffold (**Figure** **6**a). Cell–cell adhesion and relative molecule binding are positively regulated by Si in BMSCs (**Figure** **7**a–d). To simulate the moderate thermal condition constantly, BMSCs are cocultured with the scaffolds, and the coculture system is placed in the cell incubator in a 40 °C atmosphere and 5% CO_2_. Cytotoxicity assay manifests DBM and SNS@DBM scaffolds with moderate thermal stimulation have no obvious adverse influence on progenitor cells for osteogenesis like BMSCs and MC‐3T3E1 (Figure S16a,b, Supporting Information). Cell proliferation experiment demonstrates that moderate hyperthermia can promote the proliferation of BMSCs for tissue regeneration (Figure S17, Supporting Information). After recruiting BMSCs and promoting their proliferation at the bone loss lesion, SNS@DBM scaffolds induce the osteogenic gene expression with moderate thermal stimulation. The quantitative RT‐PCR arrays reveal that the SNS@DBM scaffold can upregulate the expression of osteogenic genes (Figure 6c). The western blot indicates that the protein translation of osteogenic genes is promoted in the SNS@DBM group and further augmented by moderate thermal stimulation (Figure 6b). GO analysis suggests that the SNS@DBM scaffold has a strong association with the biological processes of ossification and osteoblast differentiation in BMSCs (Figure 7a–d).

In the next period, when gene expression and protein synthesis are promoted by the SNS@DBM scaffold and moderate hyperthermia, the procedures of biomineralization and osteogenesis are launched. Alkaline phosphatase is a pivotal enzyme for the deposition of calcium salts in osteogenesis.^[^ ^58^ ^]^ BMSCs cultured with SNS@DBM scaffold show an outstanding activity in osteogenic differentiation and moderate thermal stimulation promotes this tendency in each subgroup, which can be observed through BCIP/NBT alkaline phosphatase staining (Figure 6d). Alizarin Red S staining displays the formation of calcium nodules, which is consistent with the results of qPCR arrays, WB analysis, and alkaline phosphatase staining (Figure 6d).

Through cellular and molecular experiments, the activity of the SNS@DBM scaffold of recruiting BMSCs and inducing osteogenic differentiation under moderate thermal stimulation has been certified. GO and KEGG analysis of whole transcriptome resequencing manifest that the osteogenic differentiation may be associated with the GPCR, calcium, or cytokine signaling pathway (Figure 7b,e). More precise mechanisms remain to be explored in future studies.

## 3 Conclusions

In this study, we design and fabricate a multifunctional SNS@DBM bone grafting scaffold, which works as a “thermal switch” to provide an integrated postoperative sequential thermotherapy for tumorous bone loss by exerting three levels of photothermal stimulation (i.e., strong, moderate, and nonstimulation) in different therapeutic phases. This stepwise therapeutic strategy benefits from the distinguishing and controllable photothermal property and the osteogenic biological activity of the silicified collagen scaffold. The modification is driven by the natural affinity including hydrophobic interaction and hydrogen bonds between the SNS with certain amino acids of collagen type I. Modified with SNSs, the conventional bone graft material DBM is endowed with various novel properties, such as the photothermal effect, osteoinductive effect, and immunomodulatory effect.

As to the utilization of the PTT property, this “thermal switch” smart scaffold can exert three leveled doses of thermal stimulation corresponding to three therapeutic modes for the postoperative tumorous bone defect. The temperature can rapidly reach 58 °C in vivo under the irradiation of a 1.5 W laser, and the temperature can also be maintained at 40 ± 1 °C when the “switch” is turned to a low power.

As to the osteoinductive effect, the decalcified matrix of collagen type I supplies a filling function to bone defects, which acts as an osteoconductive channel for stem cells, endothelial cells, and osteogenic precursor cells. The SNSs as efficient photothermal agents possess exceptional biocompatibility, biodegradability, and bioactivity. The degradation product silicates can recruit BMSCs, upregulate osteogenic gene expression (e.g., SPP1, OCN, and COL1A1), and promote the biomineralization procedure in coordination with moderate thermal stimulation. Bioavailable Silicon can also generate the formation of H‐type vessels, which couple with bone regeneration at six weeks in vivo.

The SNS@DBM scaffold also participates in the local immune regulation of the lesion. The implants initiate immune responses and the PTT further motivates this process in the acute period. Subsequently, the SNS@DBM scaffold cooperating with the moderate thermal stimulation downregulates the inflammation response and promotes M2 polarization within two weeks.

The bioavailability of NIR II is another vital problem that deeply influences the translational application of photothermal therapy in clinical circumstances. Compared to high‐frequency blue or ultraviolet light, near‐infrared light has better penetration and can penetrate deep into the subcutaneous tissue layer.^[^ ^59^ ^]^ NIR II at the second biowindow (1000–1700 nm) has a stronger tissue penetration and lower surface thermal effect, which helps the treatment for deep tissues become realistic. Due to the SNS@DBM scaffold has good responsiveness to NIR II, the temperature rises over 65 °C when the laser penetrates the entire skin layer and it can still reach 45 °C after the laser partially penetrates cortical bone (Figure S20, Supporting Information). When the SNS@DBM scaffold is implanted into the knee joint capsule, the temperature can be maintained at 46 °C under a 1064 nm laser of 1.5 W.

In conclusion, we explore the osteoinductivity of silicene and provide a strategy to fabricate a “thermal switch” scaffold for tumorous bone loss. We preliminarily explore the biological mechanism by which silicene can promote bone repair. Deeper mechanisms should be further investigated for the broader application of silicene.

## Conflict of Interest

The authors declare no conflict of interest.

## Supporting information

Supporting Information

Supplemental Video 1

Supplemental Video 2

## Data Availability

The authors confirm that the data supporting the findings of this study are available within the article and its supplementary materials.
