# Supplementary material for: Natural Affinity Driven Modification by Silicene to Construct a “Thermal Switch” for Tumorous Bone Loss
Source: Adv Sci (Weinh). 2024 Jul 21;11(35):2404534. doi: 10.1002/advs.202404534 (PMC11425228; doi:10.1002/advs.202404534)
Supplement: Supplementary file 1 — Supporting Information [file ADVS-11-2404534-s002.docx]

Supplementary information

**Natural Affinity Driven Modification by Silicene to Construct A “Thermal Switch” for Tumorous Bone Loss**

*Yi-Xing Chen^†a^, Yi-Ping Luo^†a^, Xiao-Dong Hou^†a^, Lei Zhang^a^, Tian-Long Wang^a^, Xi-Fan Li^a^, Zhi-Qing Liu^a^, Jin-Hui Zhao^a^, Aihemaitijiang Aierken^a^, Zhu-Yun Cai^c^, Bing-Qiang Lu^a^, Shuo Tan^a^, Xin-Yu Zhao^*a^, Feng Chen^* a, c^, Zi-Fei Zhou^*a^, Long-Po Zheng^*a, b^*

a Department of Orthopedics, Shanghai Tenth People’s Hospital, School of Medicine, Tongji University, Shanghai 200072, China.

b Shanghai Tenth People’s Hospital Chongming Branch, Shanghai 202150, China.

c Shanghai Key Laboratory of Craniomaxillofacial Development and Diseases, Shanghai Stomatological Hospital & School of Stomatology, Fudan University, Shanghai 201102, P.R. China.

d Department of Orthopedics Second Affiliated Hospital of Naval Medical University 415 Fengyang Road, Shanghai 200003, P. R. China.

**Appendix**

| Abbreviation | Full name |
| --- | --- |
| BTE  BCIP  NBT  BMSCs  CCK-8  CH3CN  COL1A1  DBM  H&E  M1  M2  MD  MT  NIR  NMP  NOS3  OCN  OPN  PDF  PDGF  PDTX  PFA  PTT  RMSD  Runx2  SNSs  SPP1  VEGF | bone tissue engineering  5-bromo-4-chloro-3-indolyl phosphate  nitroblue tetrazolium chloride  bone mesenchymal stem cells  Cell Counting Kit-8  anhydrous acetonitrile  collagen type Ⅰ alpha-1  decalcified bone matrix  hematoxylin-eosin  classically activated phenotype of macrophage  alternatively activated phenotype of macrophage  molecular dynamics  Masson's trichrome  near-infrared  1-Methyl-2-pyrrolidinone  nitric oxide synthase 3  osteocalcin  osteopontin  probability density function  platelet-derived growth factor  patient-derived tissue xenograft  Paraformaldehyde  photothermal therapy  root mean square deviations  runt-related transcription factor 2  silicene nanosheets  secreted phosphoprotein 1  vascular endothelial growth factor |

***A: Experimental Section***

**Materials and reagents.**

Calcium silicide (CaSi2, technical, powder, Sigma-Aldrich). Iodine (I2, 99.99%, Sigma-Aldrich). 1-Methyl-2-pyrrolidinone (NMP, AR, >99.0%, Sigma-Aldrich). Acetonitrile (CH3CN, anhydrous, 99.8%, Sigma-Aldrich). Deionized (DI) water used in all experiments was obtained from a water purification system (Milli-Q system, Millipore, USA). Decalcified bone matrix (DBM, Clinical level, Xiaobo Technology Development Co., Ltd, Shanghai China, Batch No. 2012.3460176CN). Collagen Ⅰ (Rat Tail, Gibco, USA). All materials and reagents were used as received.

Alpha Minimum Essential Medium (1X) + GlutaMAX-Ⅰ (α-MEM, Gibco, USA). DMEM/High Glucose Medium (HyClone, Cytiva). Fetal Bovine Serum, Qualified (FBS, Australia, Gibco). Phosphate Buffered Saline (PBS, pH 7.4 basic 1X, Gibco). Bone marrow mesenchymal stem cells (BMSCs, SD rat, Stem Cell Bank, Chinese Academy of Sciences). Human Umbilical Vein Endothelial Cells (HUVEC, Chinese Academy of Sciences). Cell Counting Kit-8 (CCK-8, Beyotime, China). BCIP/NBT Alkaline Phosphatase Color Development Kit (Beyotime, China). Alizarin Red S Staining Solution (0.2%, pH 8.3, Beyotime, China). Matrigel® Matrix Basement Membrane (Bedford, MA, USA). Glutaraldehyde (AR, 50% in H2O, Aladdin). Crystal Violet Staining Solution (0.1%, Beyotime, China).

Monoantibody from mice of OCN, OPN to rat (Santa Cruz Biotechnology INC, USA). Western Blot Kit (Beyotime, China). Trizol reagent (Ambion, Life Technologies, USA). HiScript Ⅲ RT SuperMix for qPCR (+gDNA wiper) and Taq Pro Universal SYBR qPCR Master Mix (Vazyme Biotech CO., Ltd. China). PCR Tubes (Nonpyrogenic & Rnase-/DNase-free, AXYGEN, INC. USA) and PCR Strip Tubes (Nonpyrogenic & Rnase-/DNase-free, AXYGEN, INC. USA).

CD®(SD) Rat (IGS, Beijing Vital River Laboratory Animal Technology Co., Ltd.). Balb/C nude mice (Sipur Bikai Laboratory Animal Co., Ltd). Isoflurane (Iflurin, Ringpu bio-Pharmacy Co., Ltd. China).

**Characterization.**

Transmission electron microscopy (TEM) photographs and energy dispersive X-ray spectroscopy (EDX/EDS) were collected by JEOL JEM 2100F (Japan) transmission electron microscope operated at 200 kV and JEOL ARM-300F with spherical aberration correction operated at 300 kV.

SEM photographs and the corresponding element mapping analysis were conducted on field-emission ZEISS Gemini 300 and OXFORD Xplore energy dispersive X-ray spectroscopy (EDX/EDS). The SEM photographs of biological samples were detected by Hitachi SU8010 after the pre-treatment of a critical point dryer (Leica CPD300, Germany).

Hydrodynamic particle size and zeta potential measurements were collected by Zetasizer Nanoseries (Nano AS90, Malvern Instrument). X-ray photoelectric spectroscopy (XPS, Thermo Scientific K-Alpha, USA). Fourier transform infrared spectrum (FTIR, Thermo Scientific Nicolet iS20, USA).

The optical absorption spectra were recorded on UV-1900i Shimadzu UV-Vis spectrophotometer.

The quantitative elemental analysis was detected by inductively coupled plasma-optical emission spectrometry (ICP-OES/MS, Agilent 7800 MS & 5110 OES, Agilent Technologies, USA).

The temperature variation and the corresponding thermal photograph records were collected by an infrared thermal imaging instrument (Fotric 225s camera, China). NIR laser irradiation was produced by a high-power multimode pump laser (FC-1064-5000-MM, Shanghai Xilong Optoelectronics Technology Co., Ltd).

The western blot experiment was conducted on an electrophoresis apparatus (PowerPac Basic, Bio-Rad, USA) and the real-time quantitative PCR detection was performed on LightCycler^®^ 96 instrument (Roche Diagnostics, Indiana, USA).

**Method**

**Synthesis of Silicene Nanosheets (SNSs)**

The reaction ingredient 1524 mg of I_2_ grains was resolved in 160 mL of anhydrous acetonitrile (CH3CN) under continuous stirring, then the other substrate 575 mg of CaSi_2_ was added into the reaction system. This reaction process was prolonged for 21 days under N_2_ atmosphere protection at RT. Then the product was collected by centrifugating at 4 ℃ at 13,000 rpm and the precipitate was washed with anhydrous acetonitrile and then 1-Methyl-2-pyrrolidinone (NMP) three times respectively. The product was finally dispersed in the NMP for the subsequent ultrasound exfoliation under an output power of 300 W with an ice bath. After 48h continuous ultrasound exfoliation, the mono-layer silicene nanosheets were suspended in the NMP regent stored at 4 ℃ for usage.

**Interaction between the SNSs and Collagen type Ⅰ**

The dispersant system of SNSs suspension was replaced from NMP to water first. The aqueous SNSs suspension was serially diluted to the concentrations of 2000, 1000, 500, and 250 μg mL^-1^. The same preparation procedure was applied to the collagen solution. Then the SNSs suspension was mixed with the collagen solution on a 1:1 ratio in volume (v/v). The pH value of the mixture was adjusted to around 7.4 by 0.05M NaOH (aq). The reaction concentration of SNSs and collagen Ⅰ was diluted to 1000, 500, 250,125 μg mL^-1^. The mixture was shaken in the 37℃ atmosphere for 2h. The flocculent products were freeze-dried for characterizing.

**Surface Modification of Decalcified Bone Matrix (DBM) Scaffold**

The as-synthesized silicene nanosheets were dispersed in NMP regent which is unsuitable to be a padding in the critical bone defect locations. Herein, we utilize the high affinity between the SNS and collagen type Ⅰ chain (we’ve proved in the former text) to realize the surface modification of the clinically broadly applied decalcified bone matrix scaffold. To achieve this goal, the DBM scaffold was immersed in the 2 mg mL^-1^ SNS suspension (in water) shake at 37 ℃ for 2h and refreshed the SNS reaction regent for another 2h. After being washed and dried in N_2_ air current, the SNS@DBM modified scaffold was obtained.

**Computational methods.**

The molecular structure of SNS was obtained by ChemDraw and optimized by Chem3D. The molecular structure of the collagen type Ⅰ alpha Ⅰ (COL1A1) chain was constructed by homologous reconstruction on the homologous templates of 7JJV from the RCSB PDB protein data bank. In the module MODELLER of program Modeller, the homologous protein models of COL1A1 were reconstructed under the modeling accuracy of high. The optimal conformation of the COL1A1 molecular model which includes 127 amino acids was obtained according to the Probability Density Function (PDF) value.

In the next work, we cut the long chain of COL1A1 into many fragments and executed the molecular docking procedure between the SNS and these fragments every 50 Å. The molecular docking procedure was conducted in the software Autodock. The protein COL1A1 chain was set as receptor and the SNS was set as ligand. A reliable docking site of composite system with the maximum cluster phase and optimal energy score for further analysis.

The molecular dynamics simulation analysis was conducted in the software AMBER16. The force field parameters of AmberFF99SB and gaff towards COL1A1 and SNS were set among the whole system. A 1 nm^3^ water box was constructed centered on the protein, this region maintained electrical neutrality by adding Na^+^. During the whole molecular dynamics procedure, a limiting force of -2000 kcal mol^-1^ was exerted on the COL1A1 to keep the structural stiffness. The SNS was set in a fully flexible state. Two-step energy minimization was first conducted. Then the system temperature rose from 0 ℃ to 37 ℃ through the Langevin Temperature Control Method and the pressure was raised through the Berendsen pressure control method. Finally, the bonding energy was calculated through the PME method and the average conformation of SNS-Collagen was obtained until the RMSD value reached the target.

The Gibbs free energy of the composite was calculated through MM-GBSA and MM-PBSA modules in AMBER14 and AmberTools software. The interaction mode between SNS and collagen was analyzed upon the average structure of the SNS-Collagen composite among the last 10-50 ns of the molecular dynamics simulation.

**The evaluation of SNSs encapsulation and release rate from SNS@DBM**

The SNSs modified DBM scaffolds were prepared with graded concentrations of SNSs suspension (0.125, 0.25, 0.5, 1, 2, 4, 8 mg mL^-1^). After washing and drying in the N_2_ atmosphere, the content of Silicon was detected by ICP-OES.

The prepared SNS-modified DBM scaffolds were separated into two groups, the non-NIR group and the NIR Ⅱ group. In the non-NIR group, SNS@DBM scaffolds were immersed in PBS and shaken in a 37℃ atmosphere. The 1mL PBS samples were taken out at the point of 10 min, 30 min, 60 min, 6 h, 12 h, 24 h, 48 h, 72 h for ICP test. The solution was evenly mixed before sampling, and an additional 1 mL fresh PBS would be added to keep the volume unchanged. This sampling procedure can roughly mimic the releasing behavior of continuous diluting by body fluids circulation in vivo. In the NIR Ⅱ group, the SNS@DBM scaffolds would undergo a ten-minute 1064nm NIR irradiating before sampling. The other procedures were the same as that in the non-NIR group. The SNS@DBM scaffolds were prepared in 2 mg mL^-1^ SNSs suspension. The PBS volume was adjusted according to the mass of SNS@DBM scaffolds to ensure each milligram of scaffolds was immersed in 0.1 mL PBS.

**Photothermal-conversion property of SNSs and SNS@DBM scaffold.**

The photothermal performance of silicene nanosheets (SNSs) and SNS@DBM and their powder state were acquired and analyzed by irradiation in the 96-well plate. The SNSs suspension was diluted to 2, 1, 0.5, 0.25, 0.125, and 0.0625 mg mL^-1^ and the PBS solution was set as the control. The liquid volume was 100 μL. A 1064 nm NIR Ⅱ laser of 1.5 W (with a light spot of 0.785 cm^2^) was exerted for 10 minutes, the temperature was recorded by an infrared camera. The SNS@DBM scaffold was irradiated in air and 100 μL PBS solution. The SNS@DBM scaffold was ground to powder state and irradiated in 100 μL PBS solution. The SNS@DBM scaffold was prepared in SNSs aqueous suspension of different concentrations of 2, 1, 0.5, and 0.25 mg mL^-1^. The DBM scaffold was set as a control. A 1064 nm NIR Ⅱ laser of 1.5 W (with a light spot of 0.785 cm^2^) was exerted for 10 minutes, the temperature was recorded by an infrared camera. Different power of 1064 nm NIR Ⅱ laser was exerted on the SNS@DBM prepared in 2 mg mL^-1^ SNSs suspension for 10 minutes. The output power was adjusted to 1.5, 1, 0.75, and 0.5 W (with a light spot of 0.785 cm^2^), and the temperature was recorded by an infrared camera.

**Cell viability assay.**

The bone mesenchymal stem cell line (noted as BMSCs were kindly provided by Stem Cell Bank, Chinese Academy of Sciences) and the pre-osteoblast MC-3T3 E1 cell line were cultured at 37 ℃ under 5% CO2 in α-MEM (α-MEM, GlutaMax, Gibco, Invitrogen) supplemented with 1% penicillin/streptomycin (Gibco, Invitrogen), and 10% Australia fetal bovine serum (FBS, Gibco, Invitrogen) in a humidified incubator.

BMSCs and MC-3T3 E1 cells were prepared for the biocompatibility trial in *vitro* study. Briefly, the stem cells and MC-3T3 were incubated in a culture flask (Nest, China) and allowed to develop to logarithmic growth period. Then the cell lines were harvested by treatment with 0.05% (0.25% for MC-3T3 E1) trypsin-EDTA solution (Gibco, USA). Then the cell lines were seeded into 96-well plates at a density of 1 × 10^4^ cells/well in a completed α-MEM medium at 37 °C and 5% CO_2_ for 24 h adherence. The in *vitro* cytotoxicity of SNS@DBM (2 mg mL^-1^ SNSs suspension modified) scaffolds and the NIR intervention was evaluated by a standard Cell Counting Kit-8 (CCK-8) viability assay of BMSCs and MC-3T3 E1 cells. In detail, the DBM and SNS@DBM scaffolds were put in 96-well plates to coculture with the cell lines for 24 h. The blank group without any treatment is as a control. Then a 1064nm NIR irradiation at 0.75W output power for 10min was conducted on the NIR group. Then the CCK-8 viability assay was applied to evaluate the cell viability (n=4).

The viability and morphology of BMSCs on the micro-surface of SNS@DBM were further validated by SEM photographs. The BMSCs were seeded into the scaffolds of DBM and SNS@DBM. After 48 hours of culture for attachment, the cell-scaffold coculture system was fixed by 5% Glutaraldehyde and dehydrated by 10%, 20%, 30%, 50%, 70%, 80%, and 95% alcohol. Then the sample which is at a critical drying state was prepared for SEM (n=3).

***In vitro* photo-induced tumor cells apoptosis.**

The human osteosarcoma cell line 143b cells were first seeded in 96-well plates at a density of 1 × 10^4^ cells per well in DEME High Glucose Medium at 37 °C in the presence of 5% CO2 for 24 h before treatment. Then the SNS@DBM (2 mg mL^-1^ SNSs suspension modified) scaffolds were added to the wells. These wells containing SNS@DBM scaffolds and cocultured cells were then irradiated for 10 min under a 1064 nm laser at varied output power (0, 0.5, 0.75, 1.0, and 1.5 W). Another experiment was done under the invariant laser output power of 1.5 W of a 1064 nm laser but the duration varied (0, 1, 2, 3, 4, and 5 min). Finally, a standard CCK-8 assay was used to evaluate the viability of cells (n=4).

**Photothermal therapy for PDTX tumor model.**

First, the tumor tissue was harvested from the patient who was diagnosed with osteosarcoma and performed a tumorectomy surgery (Ethics approval NO. SHSY-IEC-5.0/22k46/P01). Then the tumor tissue was divided into several parts around 1 mm^3^ for each piece, to inoculate on the back of nude mice subcutaneously. Once the tumor was growth up to 1000 mm^3^, the tumor would be harvested and the tumor grafting procedure would be repeated. Experiencing one or two generations of development, the tumorigenicity and homogenization were at a steady situation.

One week before the treatment, the PDTX tumor model was established on nude mice. The 4~5 weeks-old nude mice were separated into 6 groups randomly (Blank, NIR only, DBM, DBM + NIR, SNS@DBM, SNS@DBM +NIR). Then the prepared tumor tissue pieces were embedded being mixed with the powder state of the DBM and SNS@DBM scaffolds on the back of nude mice hypodermically. In the blank control group, the tumor pieces were embedded alone (n=6). During around one week’s growth, once the tumors’ volume reached ~150 mm^3^, the photothermal therapy of a 1064 nm NIR of 1.5 W output power (with a light spot of 0.785 cm^2^) was performed on the package blot on the back for 10 min. The temperature and heat images were recorded by an infrared camera. The hyperthermia treatment was conducted only once on the first day of the experimental duration. All mice were anesthetized well before NIR laser treatment. The tumor volume was measured by a digital caliper every two days and the photo of the tumor growth was taken every 3 days *in vivo* during the detection of 28 days. The tumor volume was measured according to the following formula: tumor volume = (tumor length) × (tumor width)^2^ /2. The tumors and organs were harvested at the end of the detection and fixed in 4% paraformaldehyde (PFA). Subsequently, the tumor tissues were taken digital photos and stained with H&E and Ki-67 for pathological analysis. The organs from different groups were stained with H&E for histological analysis. Mice with tumors larger than 1500 mm^3^ or tumor lengths longer than 20 mm should be euthanized according to the standard animal protocol.

***In vitro* osteogenesis property of SNS@DBM scaffolds and mild-heating treatment.**

The bone mesenchymal stem cell line was applied to explore the ability of osteogenesis of the SNS@DBM scaffolds and mild-heating treatment. BMSCs were cultured at 37 ℃ under 5% CO2 in α-MEM supplemented with 1% penicillin/streptomycin, and 10% Australian fetal bovine serum in a humidified incubator. When the BMSCs were growing rapidly at the logarithmic growth period, the cells were seeded into the cell plate for 24-hour adaptation. When the cell density was up to 80%, the DBM and SNS@DBM scaffolds were cocultured with the cells in the up-well of a trans-well chamber, and the medium was replaced with the fresh-induced medium. The blank group where BMSCs were cultures in an induced medium without any other treatments was as a control. To maintain the mild-heating environment and explore its effects on the osteogenic differentiation of BMSCs, we set up a subgroup among the cocultured system. One group was cultured at 40 ℃ under 5% CO2 and in the other group, the culture condition didn’t have any change.

After one week’s culture, some BMSCs in the wells were fixed in 4% PFA and stained with a BCIP/NBT kit for the test of alkaline phosphatase activity (n=3). The other BMSCs were collected for real-time quantitative PCR of osteogenic genes OCN, SPP1, Runx2, and COL1A1 and western blot of corresponding protein of OCN and OPN (n=4). The qPCR and WB experiments were repeated three times respectively. The BMSCs which were cultured for 21 days were fixed in 4% PFA and stained with Alizarin Red S Staining Solution for the test of mineralized nodules (n=3).

***In vivo* capacity of bone repair promotion of SNS@DBM scaffolds and mild-heating treatment.**

The *in vivo* experiment of bone repair was conducted on the rat critical cranial defect mode (Ethics approval ID. SHDSYY-2021-2530). The 6-week-old rats were all anesthetized well with 3% pentobarbital sodium, then the head skin was incised and blunt separation of the tissue was until the skull was exposed well. The periosteum needs a neat cut and careful protection. A critical defect of a 5 mm-diameter round was made on the two symmetrical sides of the middle cranial suture. Then the sterilized DBM and SNS@DBM scaffolds were implanted into the defect area then the periosteum was reset before the suture. In the blank group, rats without any filler were used as a control. The subgroups of NIR and non-NIR were set among all groups of Blank, DBM, and SNS@DBM. The temperature and heat image were recorded by an infrared camera and the temperature was controlled at 40 ℃ ± 1 ℃ which is considered as a reparative temperature, under the monitoring of the infrared camera. All of the rats were intraperitoneally injected with antibiotics of cefuroxime q.d. continuously for three days to prevent the potential infection.

After 3, 6, and 12 weeks, the crania were harvested after euthanasia. The samples were fixed in 4% PFA and scanned by micro-CT for mineralization analysis in the defect area. Then the crania were detected with H&E staining, Masson staining, immunohistochemical staining of Runx2, and immunofluorescence staining of H-type vessels, and the samples for M2 polarization detection were harvested at the first and second week (n=4).

Before the euthanasia, all rats underwent cardiac blood collection for serological liver and kidney function tests (n=4).

**Statistical analysis.**

Data are shown as the mean ± standard deviation (SD). The samples or mice per group in each study are marked in the related experimental methods (*n*). Student’s t-test was applied in two samples and one-way analysis of variance (ANOVA) was applied in multiple samples to evaluate the data with different significance levels (* p <0.05, ** p <0.01, and *** p <0.001).

***B: Supplementary figures, tables and discussions***


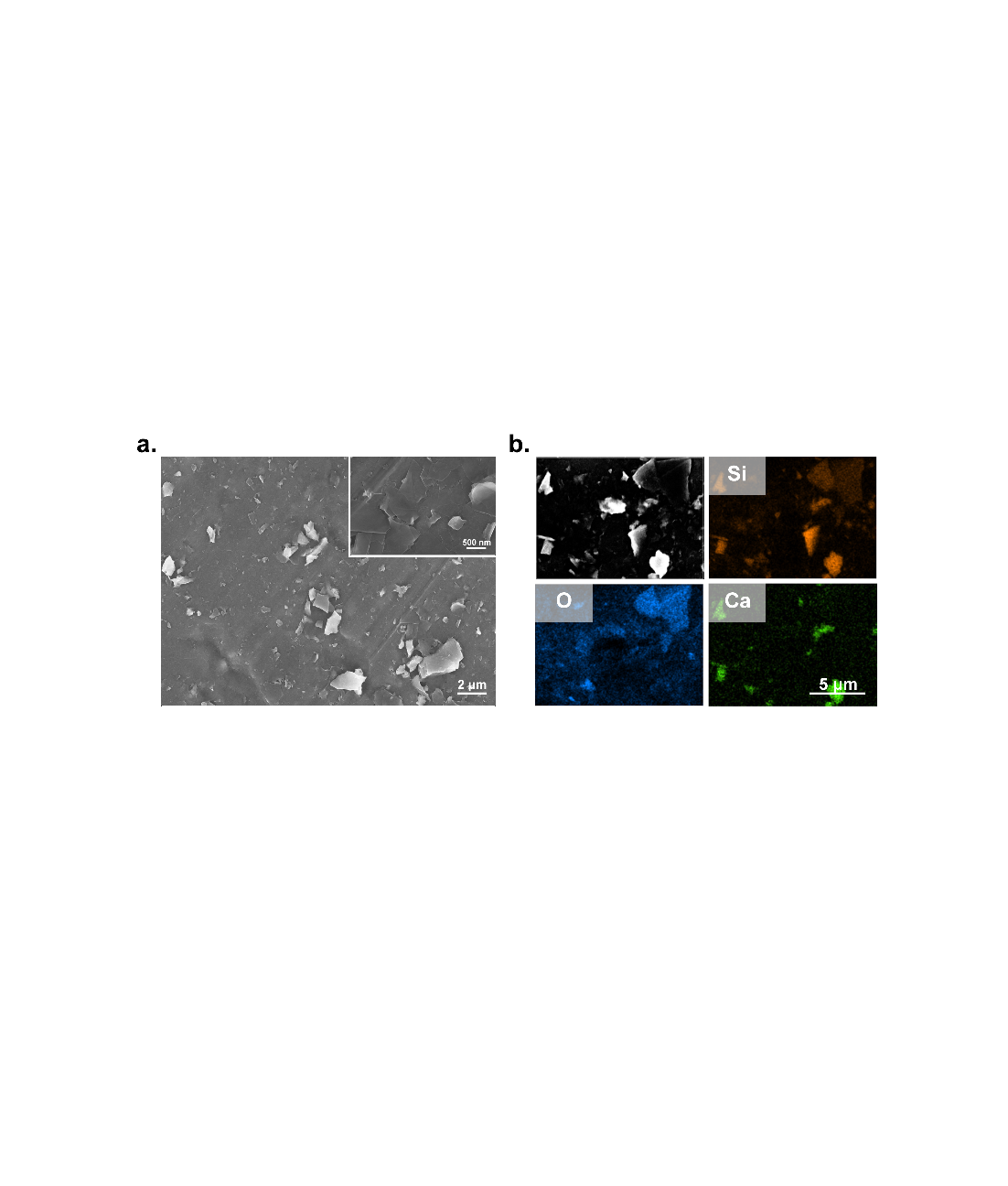


**Figure S1.** a). SEM image and b). elemental mapping image of the SNSs.


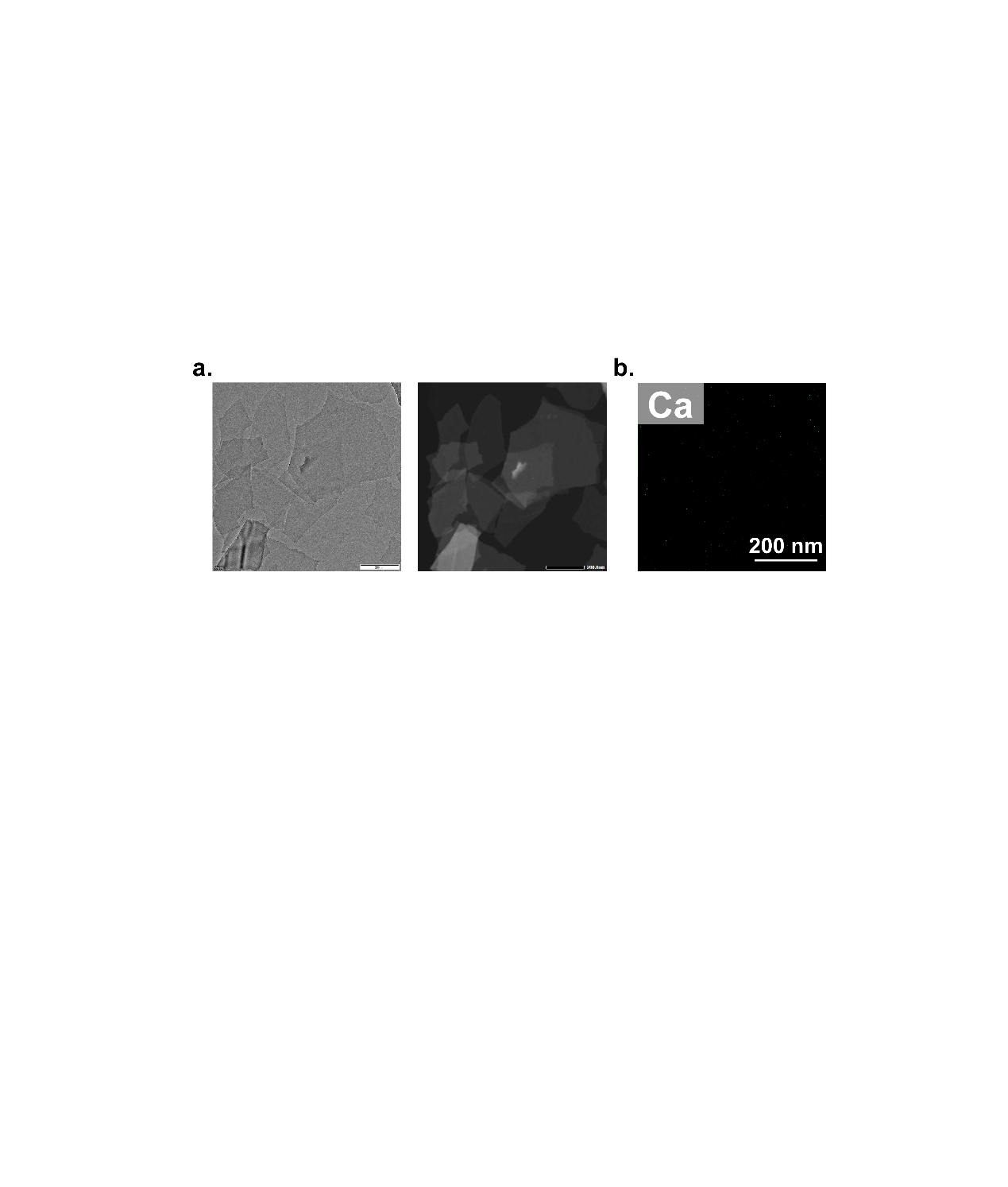


**Figure S2.** a). Bright-field and dark-filed TEM images and b). elemental mapping image of TEM image.


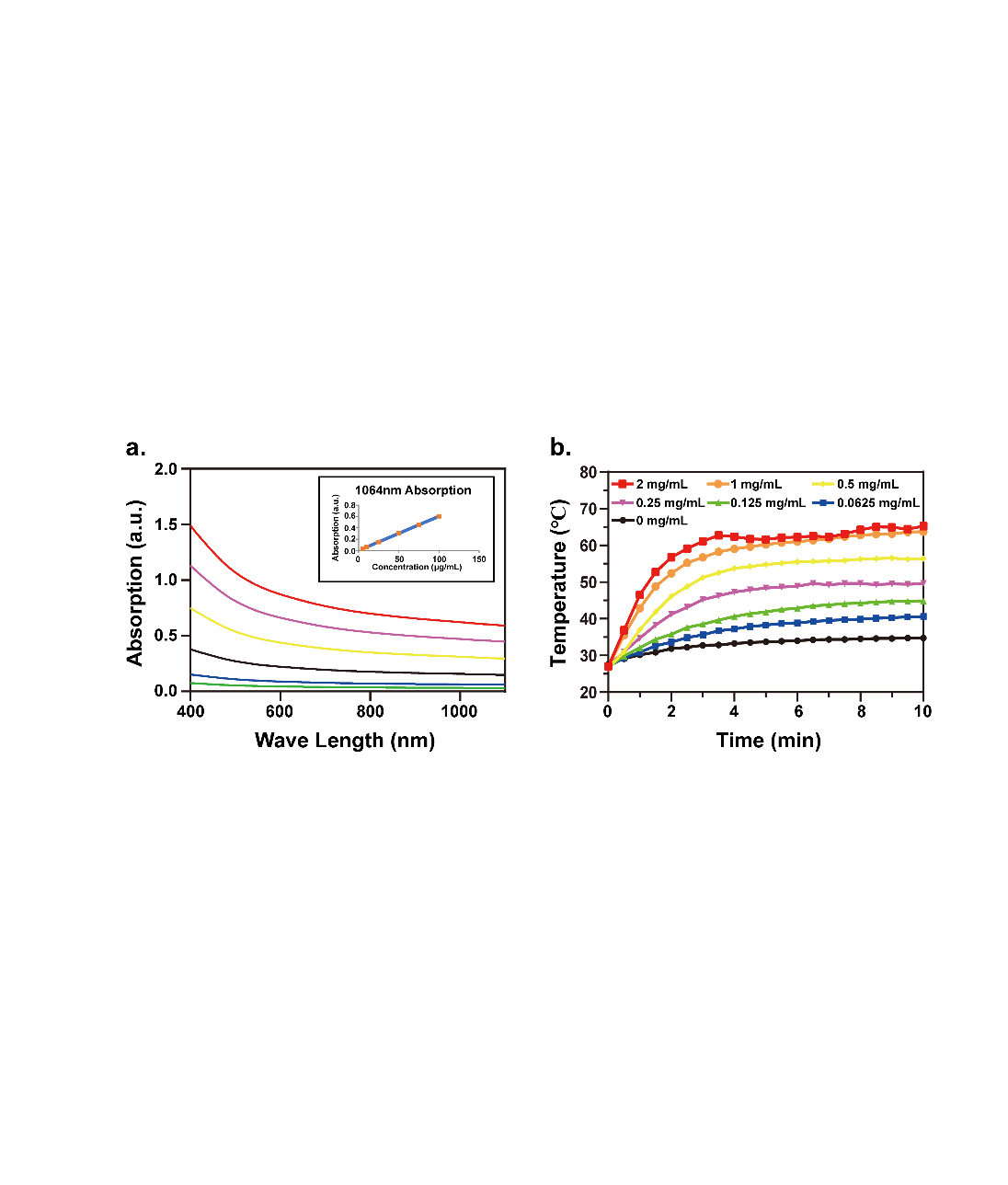


**Figure S3.** a). Absorbance spectra of silicene dispersed in water at varied concentrations of silicene (from top to bottom: 100, 50, 25, 12.5, 6.25 and 3.125 µg mL−1). Inset in a): Mass extinction coefficient of silicene at 1064 nm. b). Photothermal-conversion heating curves of silicene-dispersed aqueous suspension under NIR II laser irradiation (1064 nm, 1.5 W) at varied concentrations (0, 62.5, 125, 250, 500, 1000, and 2000 µg mL−1).


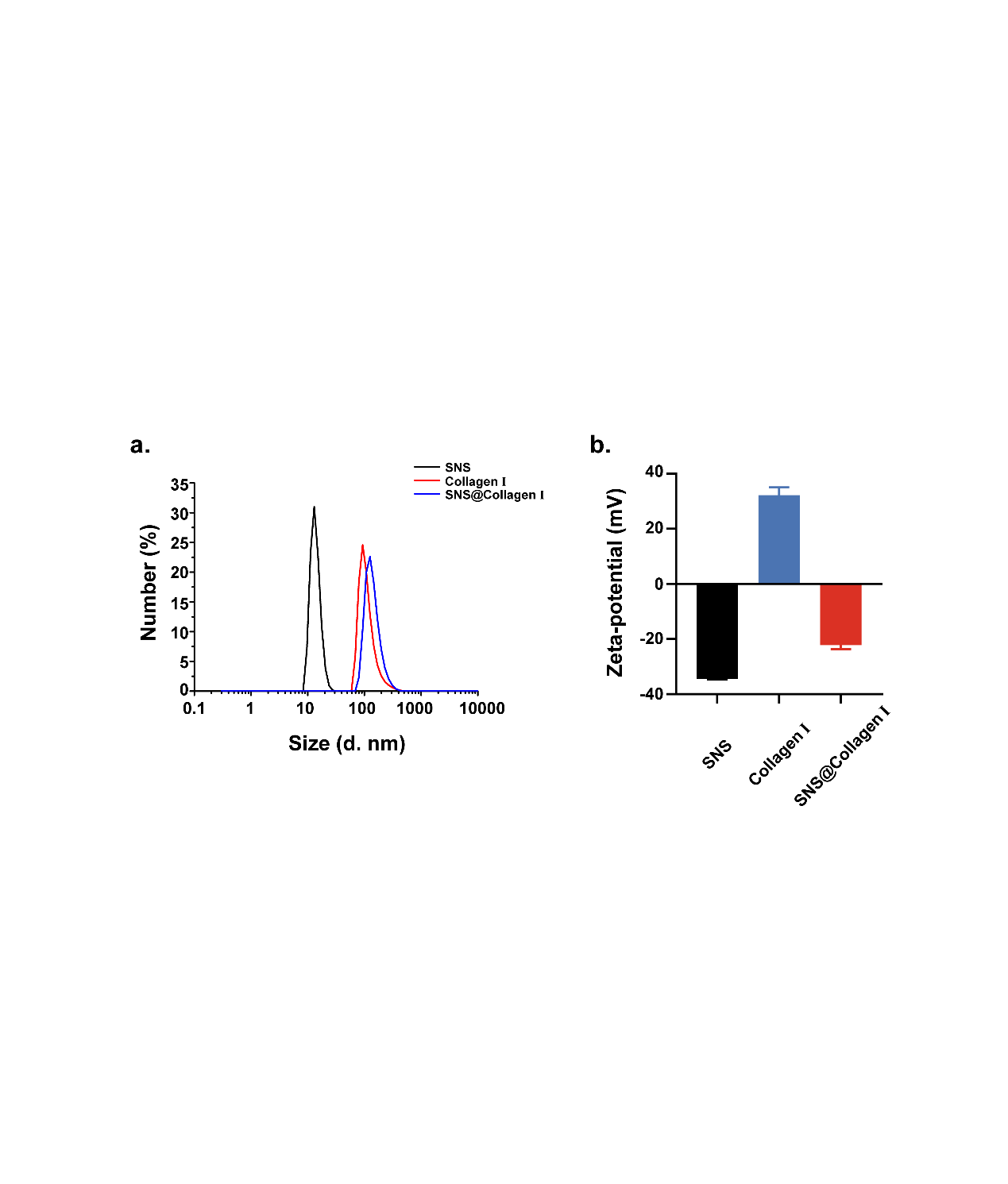


**Figure S4.** a). Hydrated particle size and b). zeta potential of SNSs, Collagen type Ⅰ and SNS@Collagen composite. (Mean ± SD, n=3)


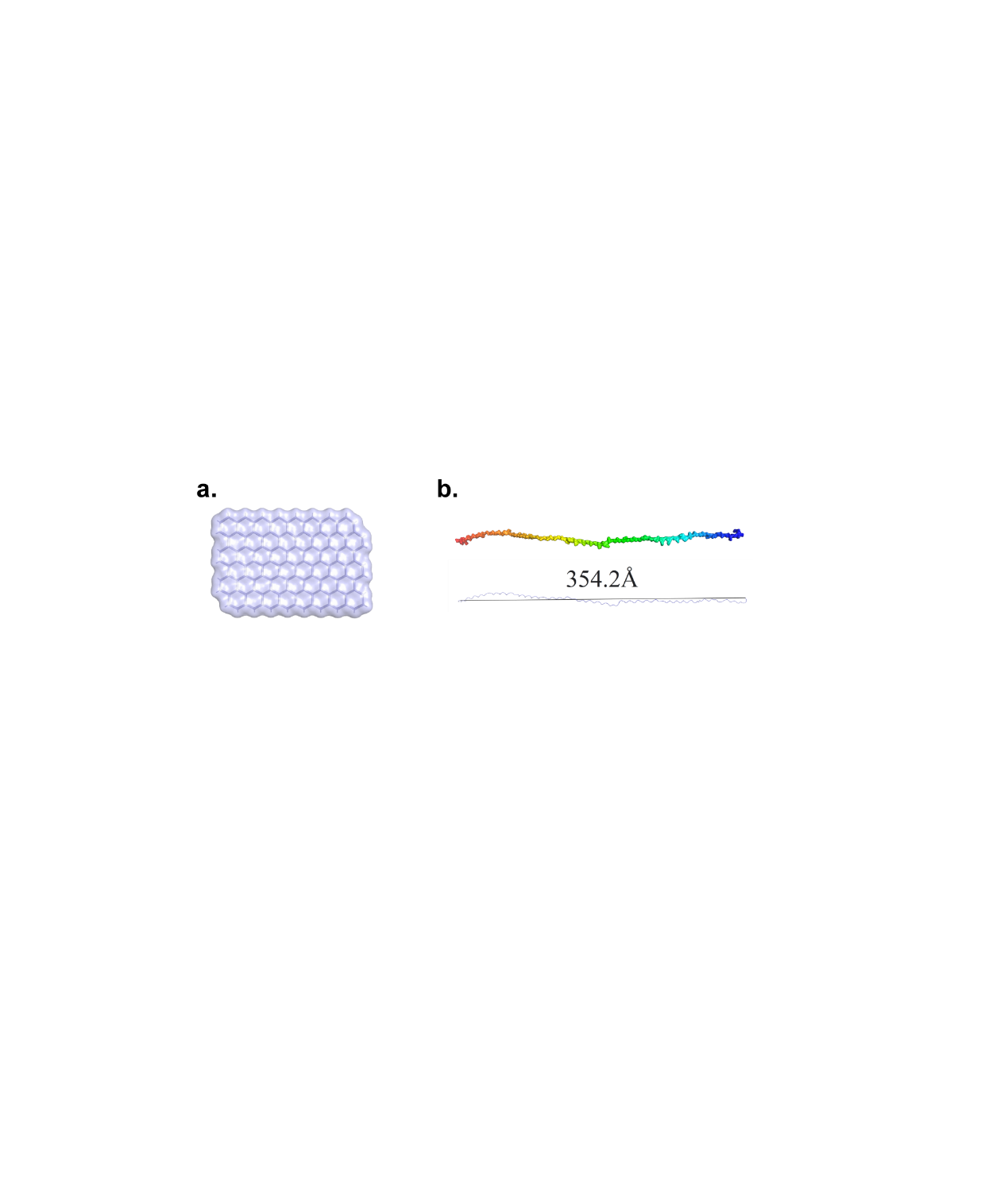


**Figure S5.** Optimized molecular structure of a). SNS and homologously reconstructed b). COL1A1 chain.


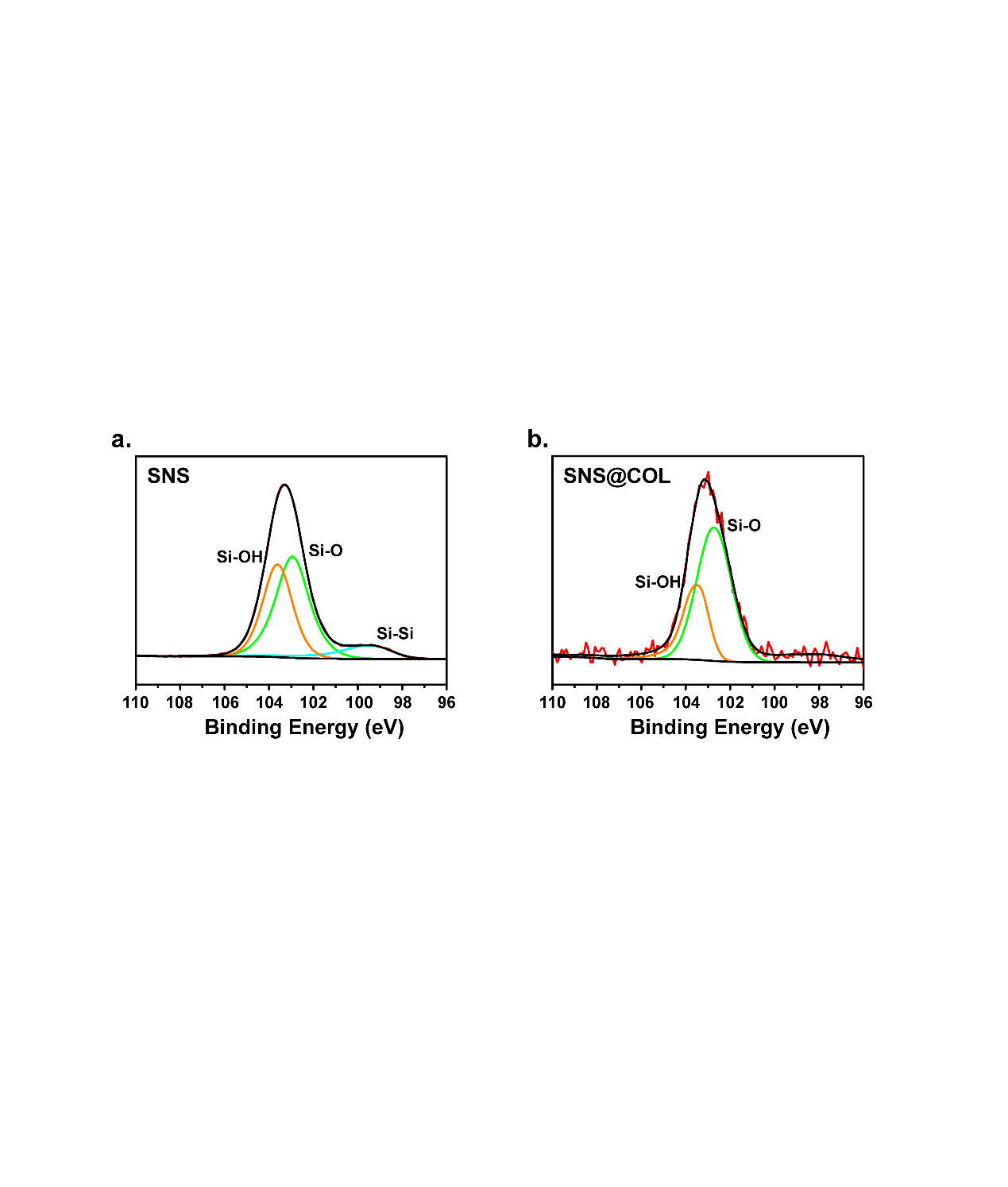


**Figure S6.** XPS analysis of silicene Si 2p region in a). SNS and b). SNS@COL composite.


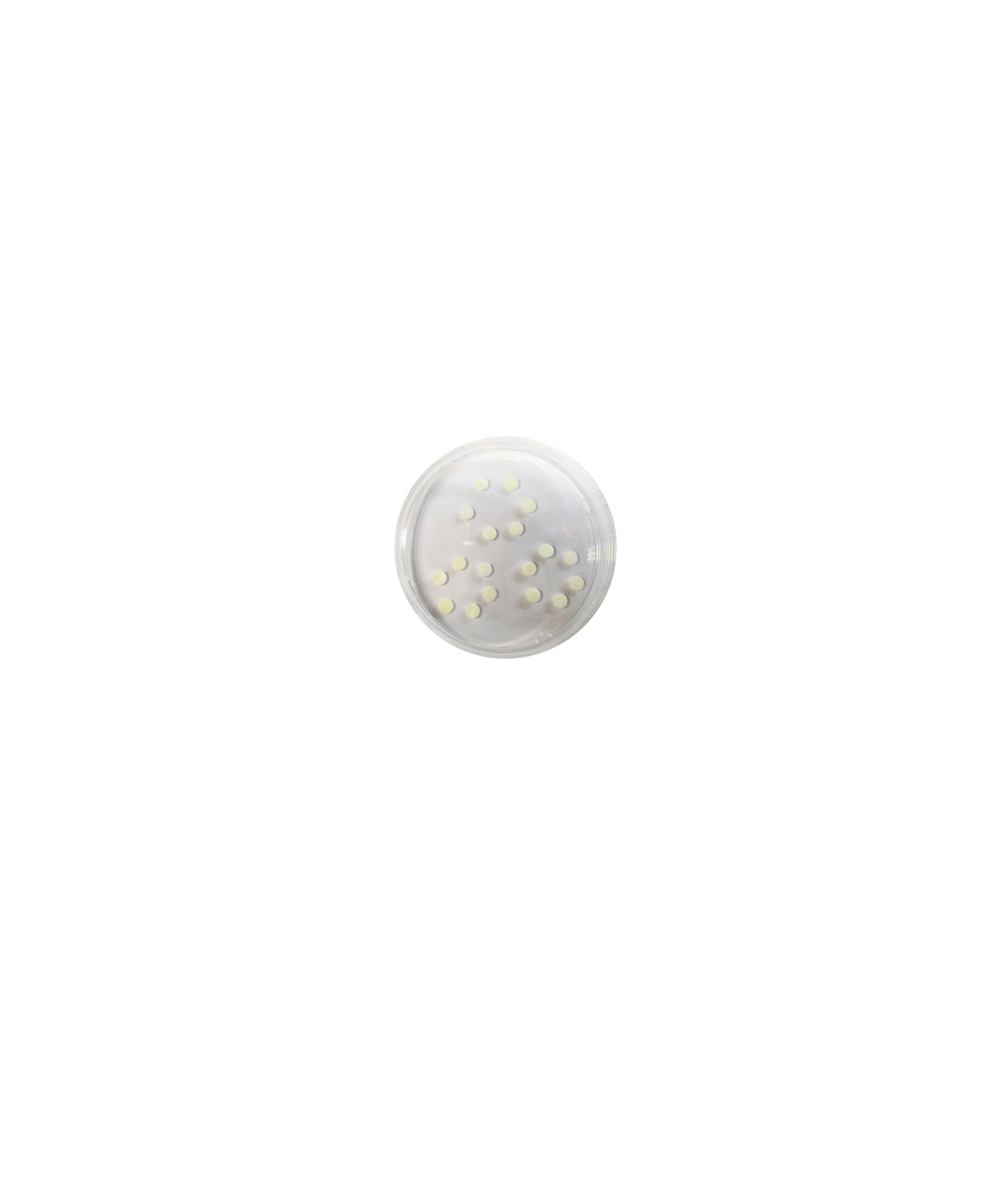


**Figure S7.** Collagen template scaffolds fabricated from decalcified bone matrix.


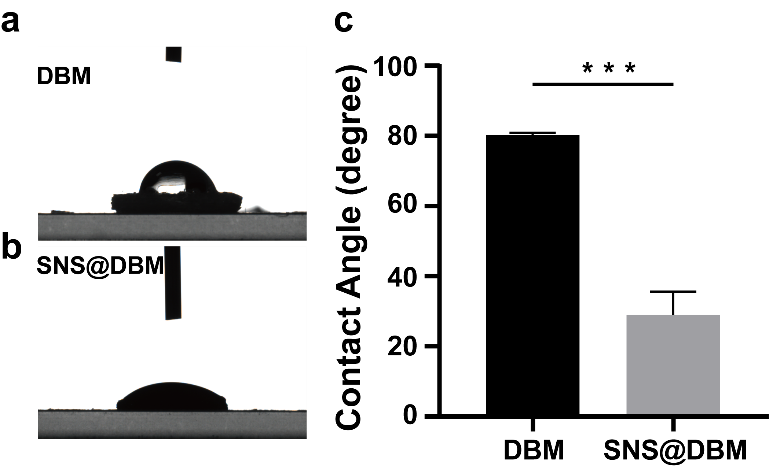


**Figure S8.** Contact angle measurement of the water droplet on a). the DBM scaffold (in a stable state) and b). the SNS@DBM scaffold (on the touching moment). c). The quantitative data of contact angles in the DBM scaffold and SNS@DBM scaffold group. (Mean ± SD, n=3)


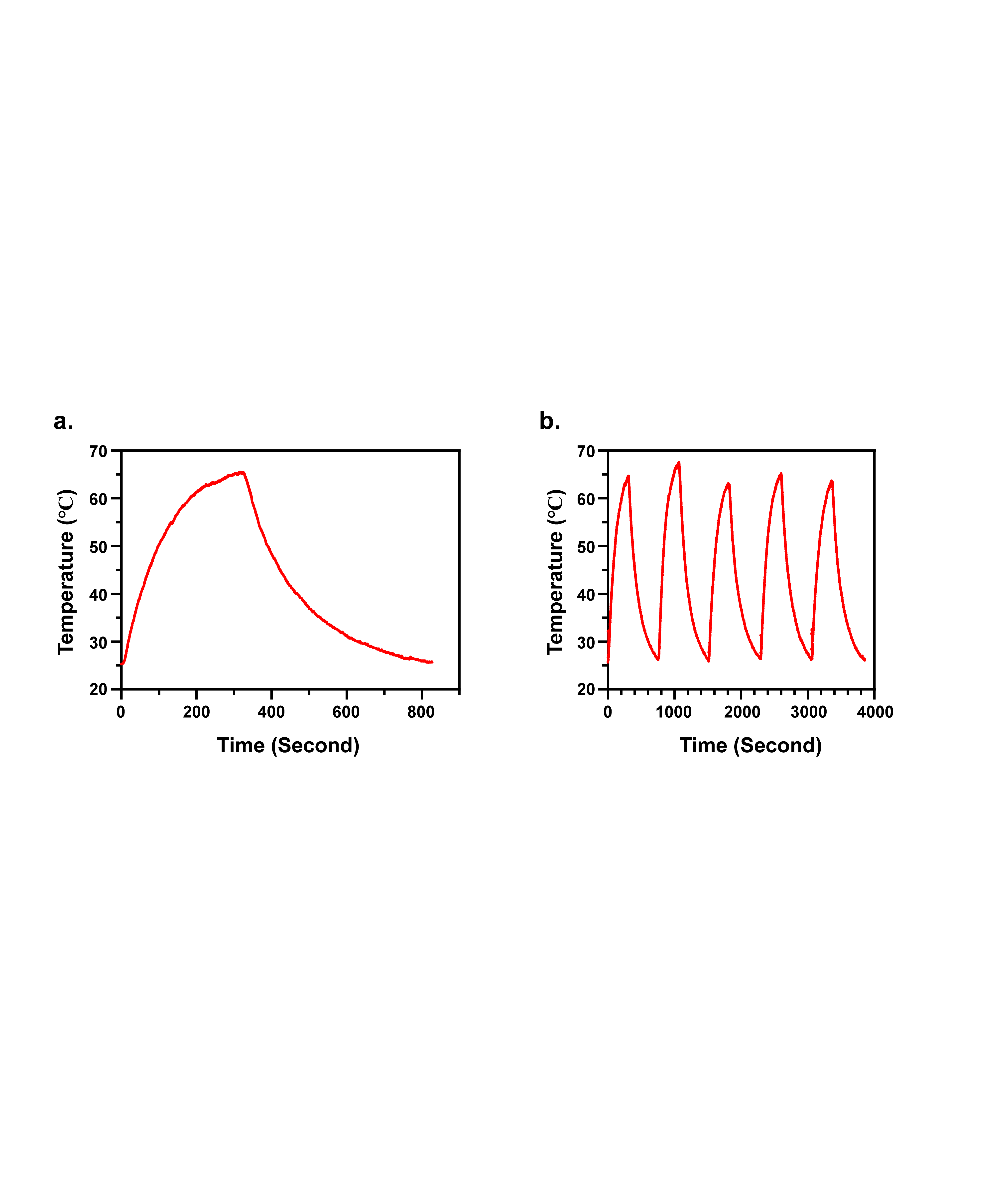


**Figure S9.** Stability of photothermal property. a). One heating-cooling cycle and b). five heating-cooling cycles with a 10-mins irradiation under a 1.5 W 1064 nm NIR Ⅱ laser.


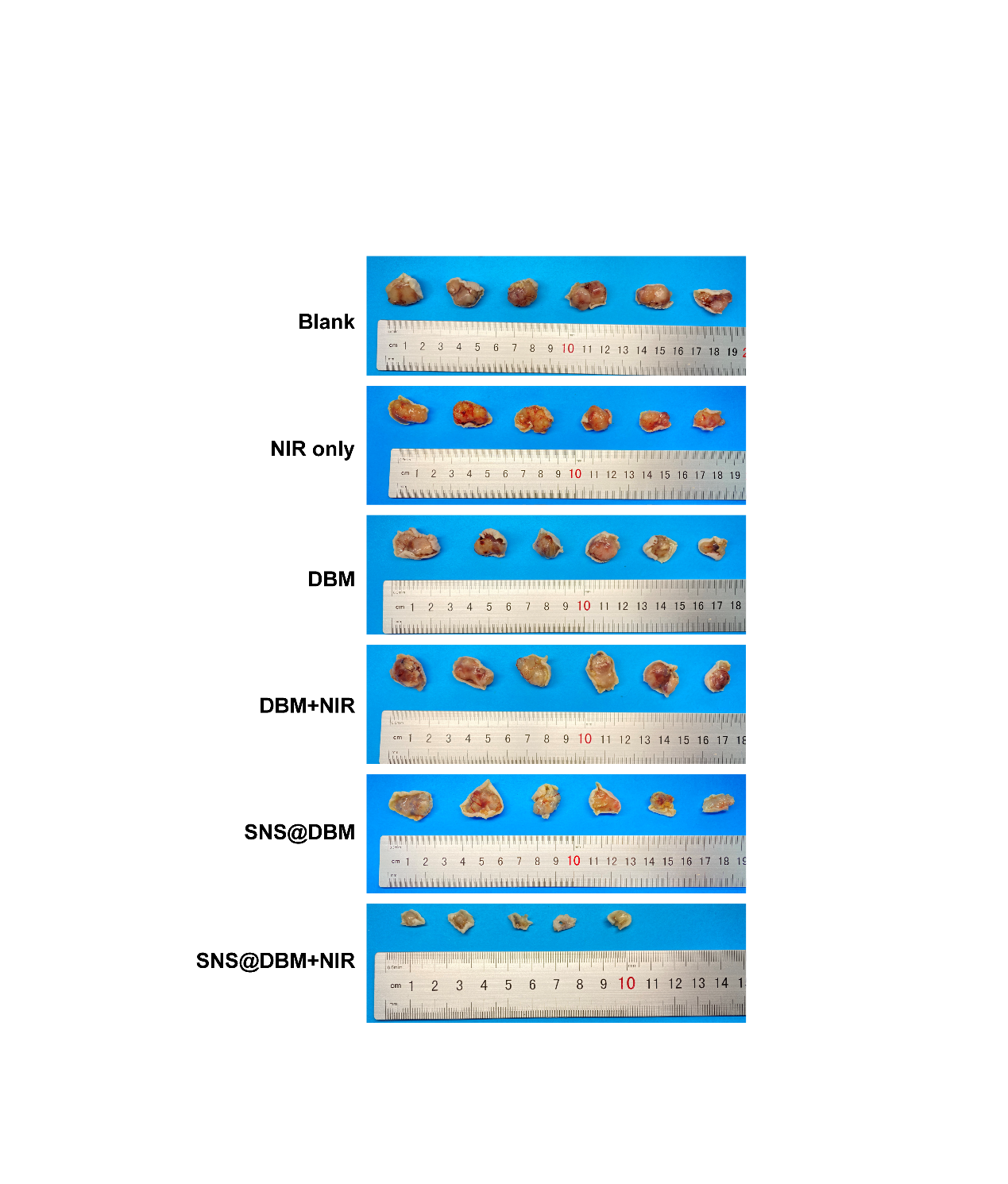


**Figure S10.** Digital photographs of isolated tumors from different treatment groups.


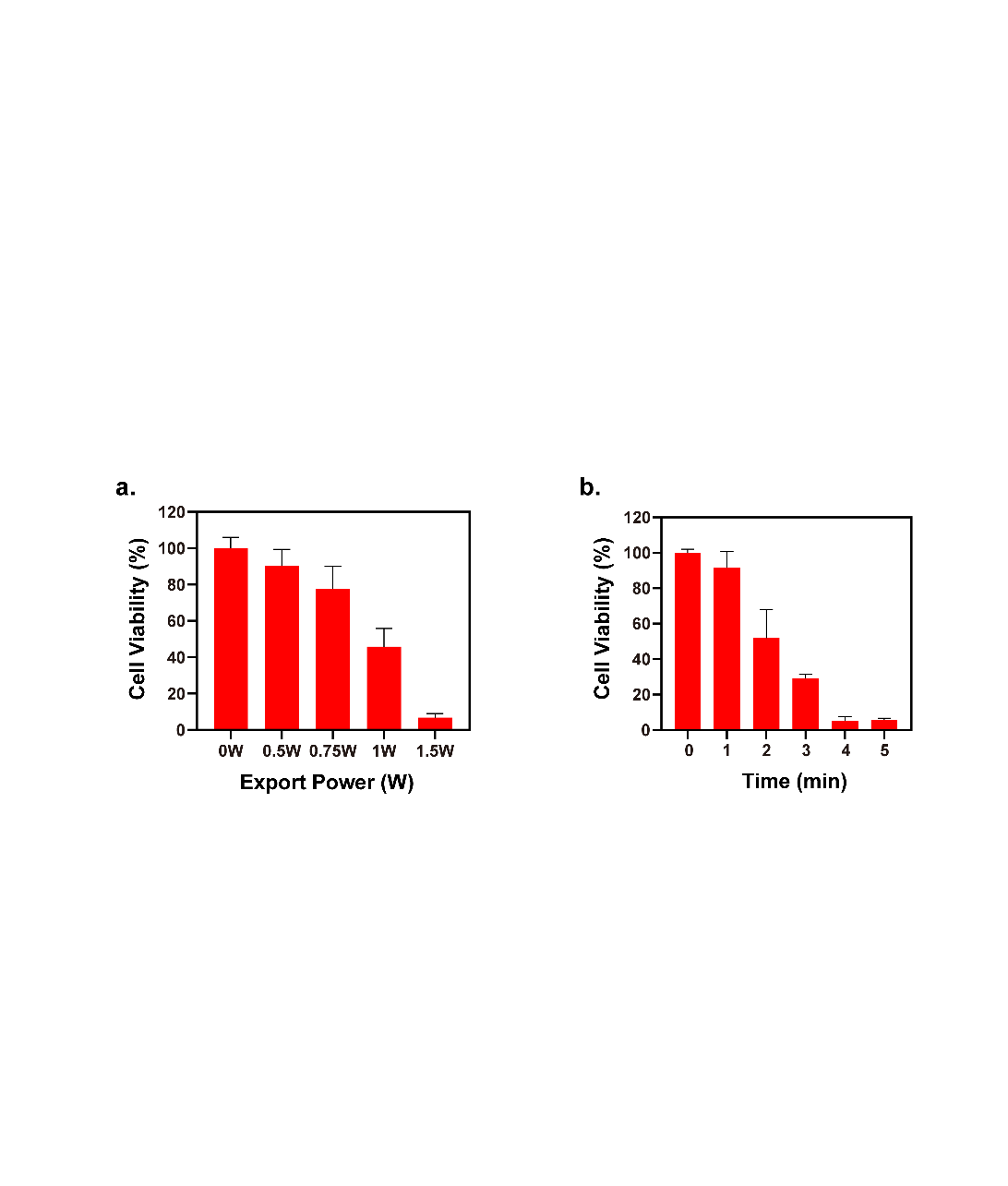


**Figure S11.** In vitro PTT efficiency of different a). output power and b). ablation time upon 143b cell lineage. (Mean ± SD, n=4)


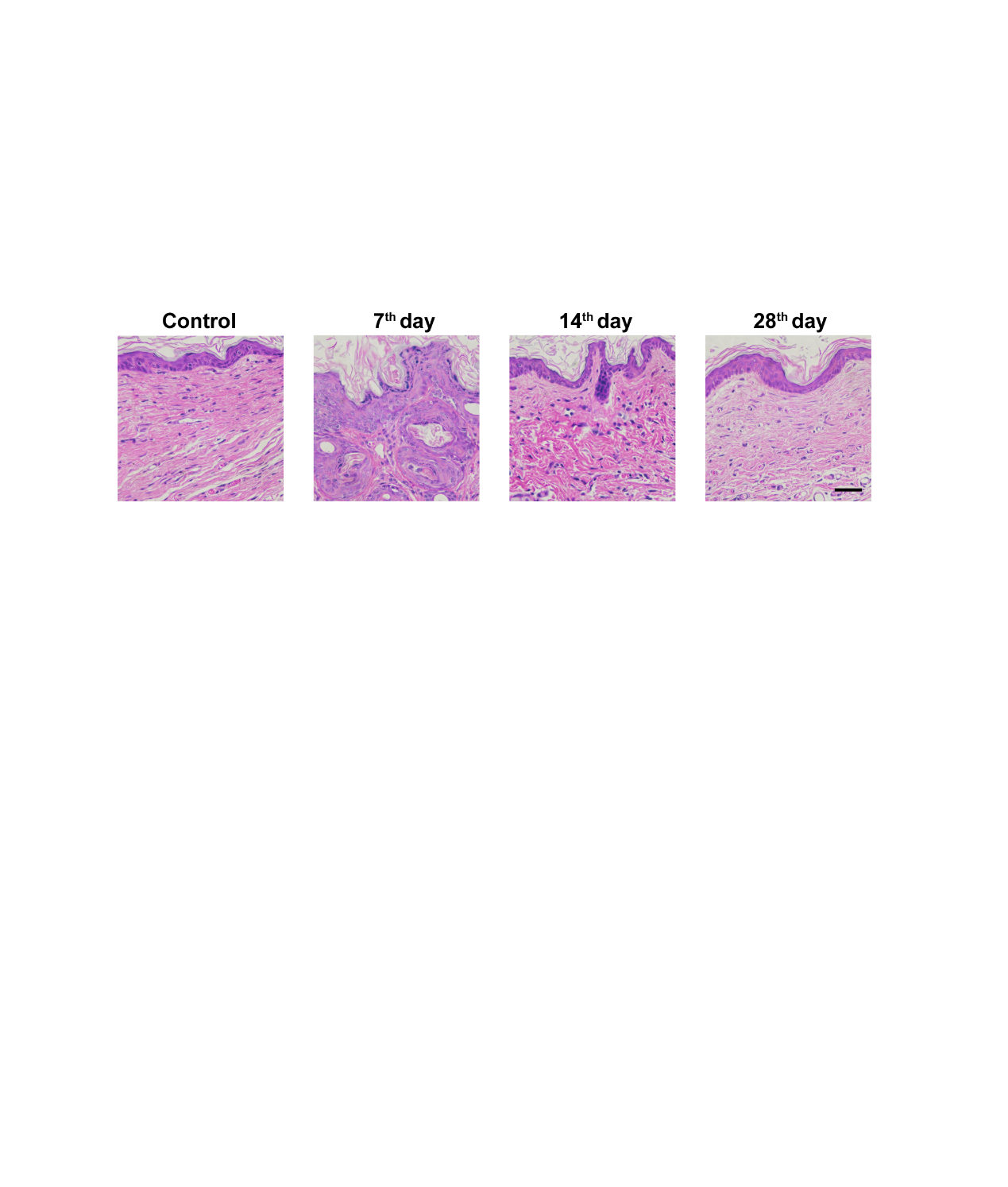


**Figure S12.** Cutaneous injury and recovery stages after in vivo PTT tumor ablation.


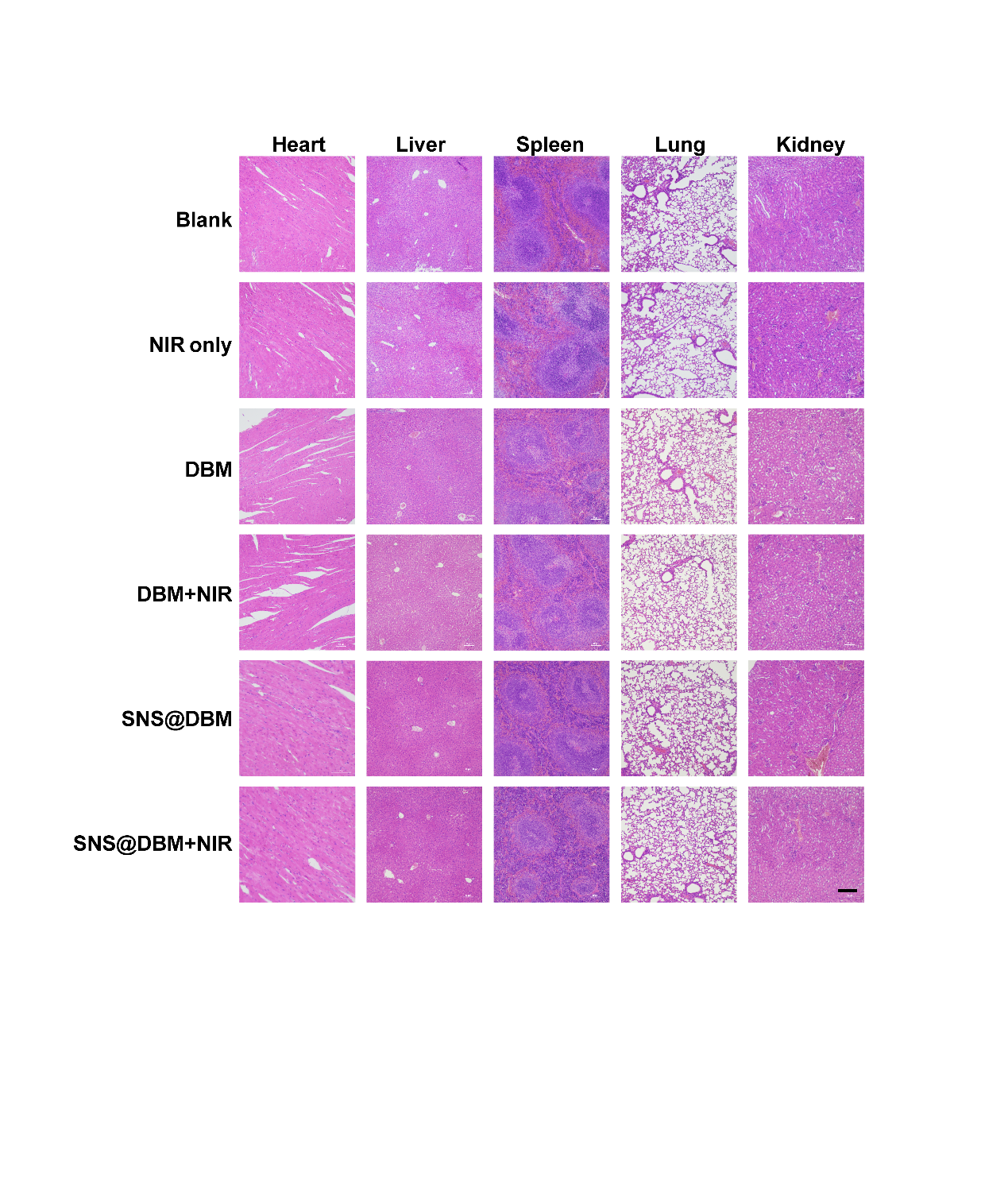


**Figure S13.** Histological morphology of major organs from different treatment groups after in vivo PTT tumor ablation.


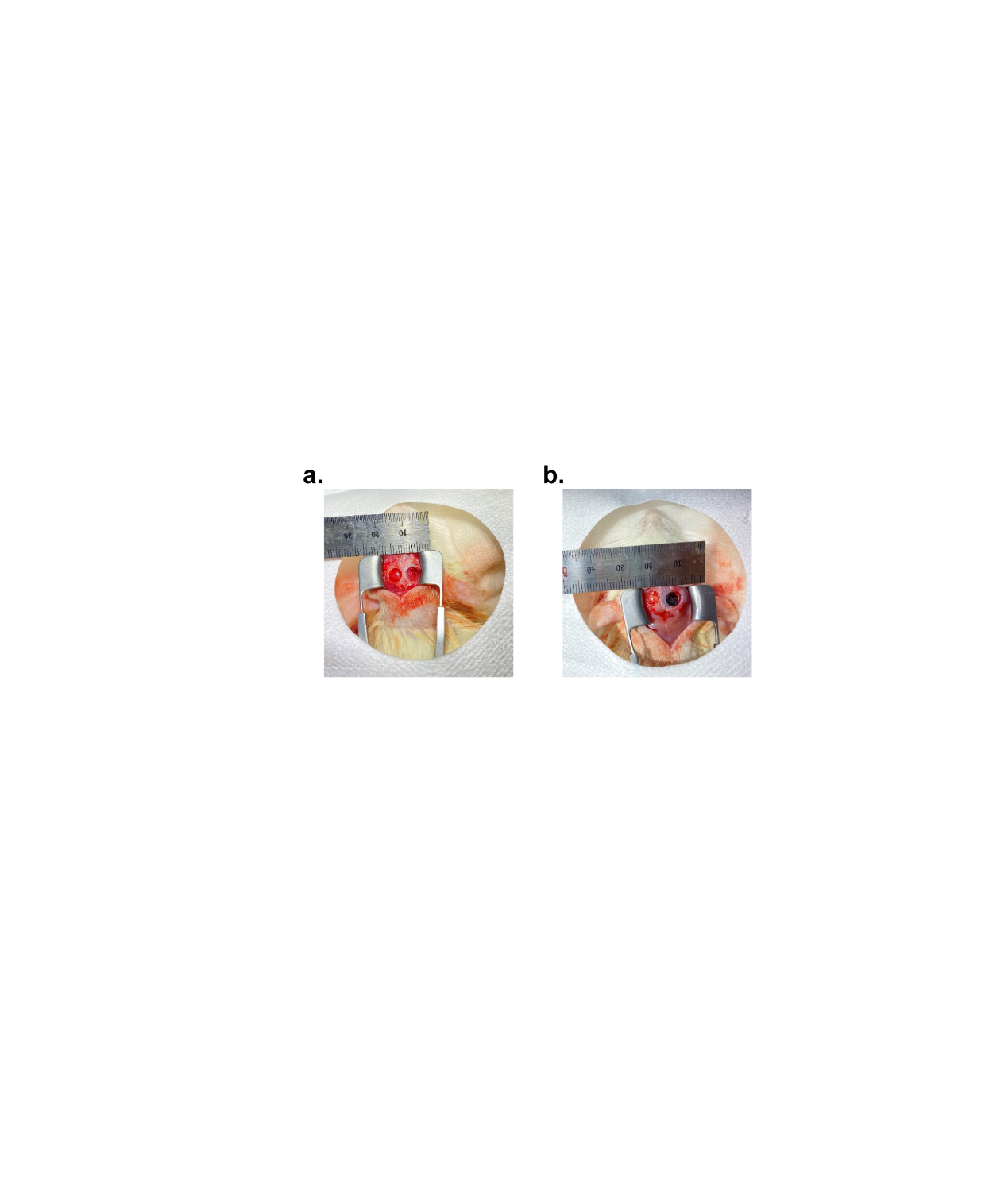


**Figure S14.** Digital photographs of rat critical cranial defect and implantation of a). DBM and b). SNS@DBM scaffold.


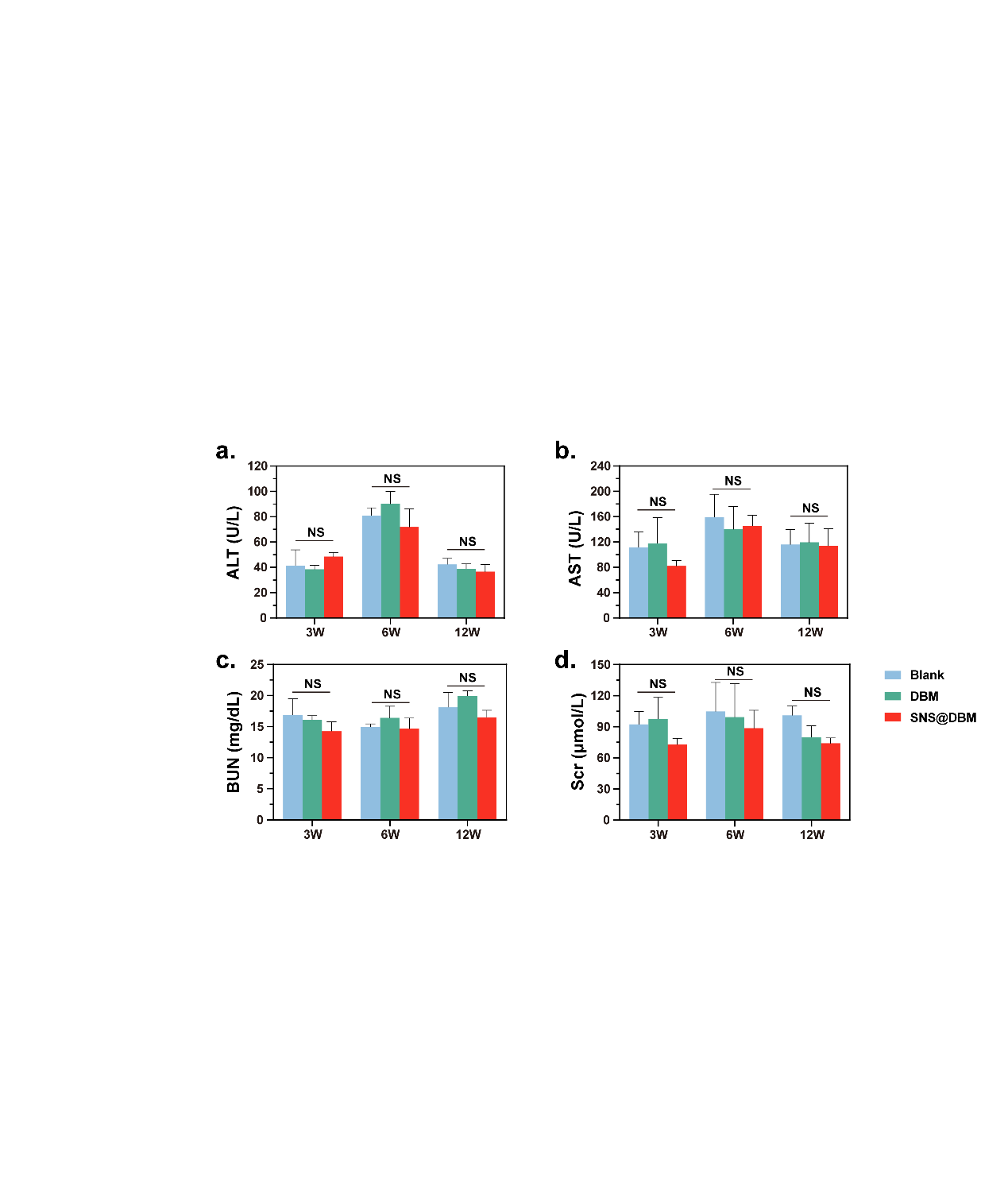


**Figure S15.** Serum biochemical indicators of liver and renal function from different treatment groups. a). Alanine aminotransferase (ALT); b). Aspartate aminotransferase (AST); c). Blood urea nitrogen (BUN); d). Serum creatinine (Scr). (Mean ± SD, n=4)


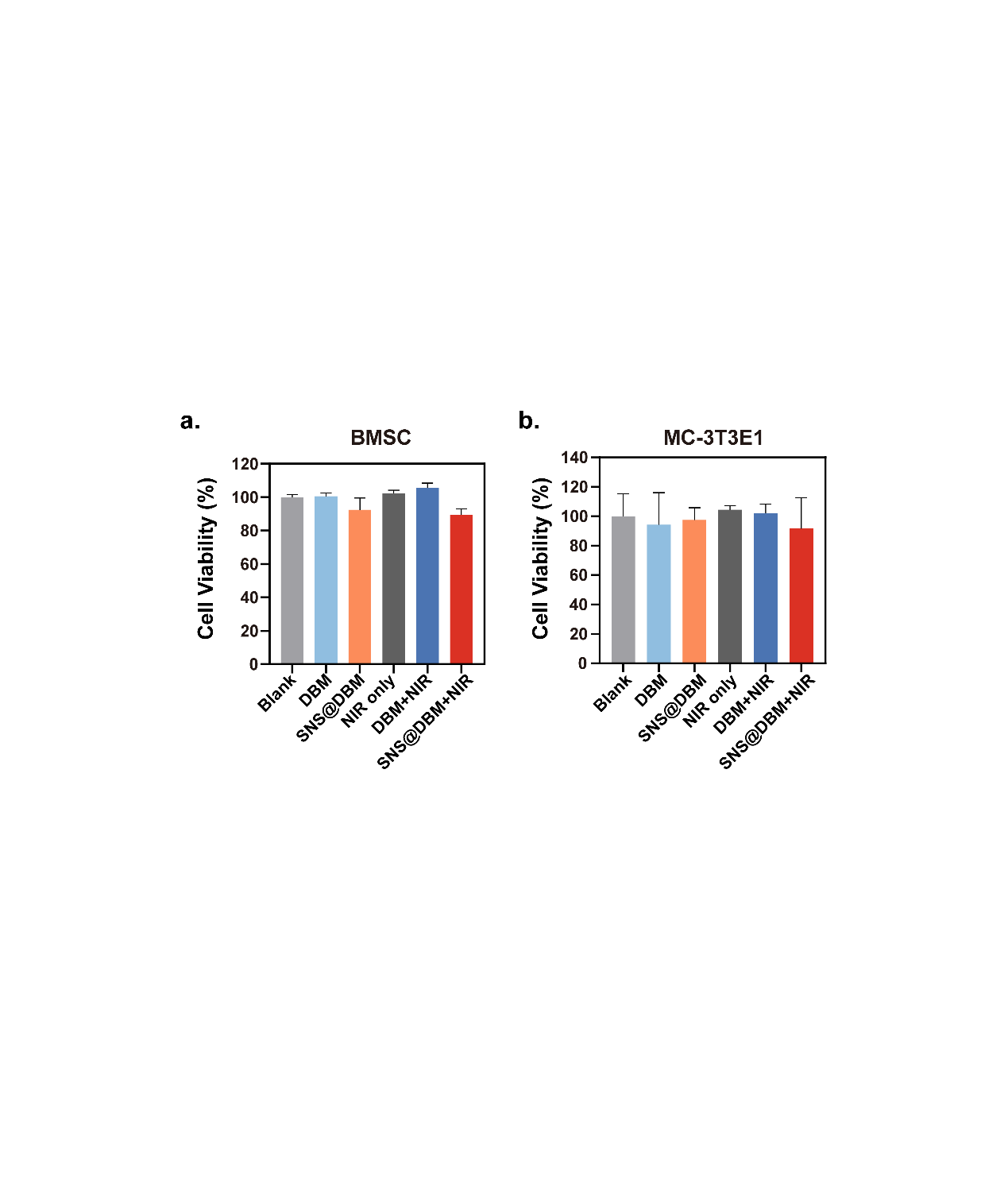


**Figure S16.** Cytotoxicity of different treatments with a 1064nm NIR Ⅱ of 0.5 W output power on a). BMSCs and b). MC-3T3E1 cell lineage. (Mean ± SD, n=4)


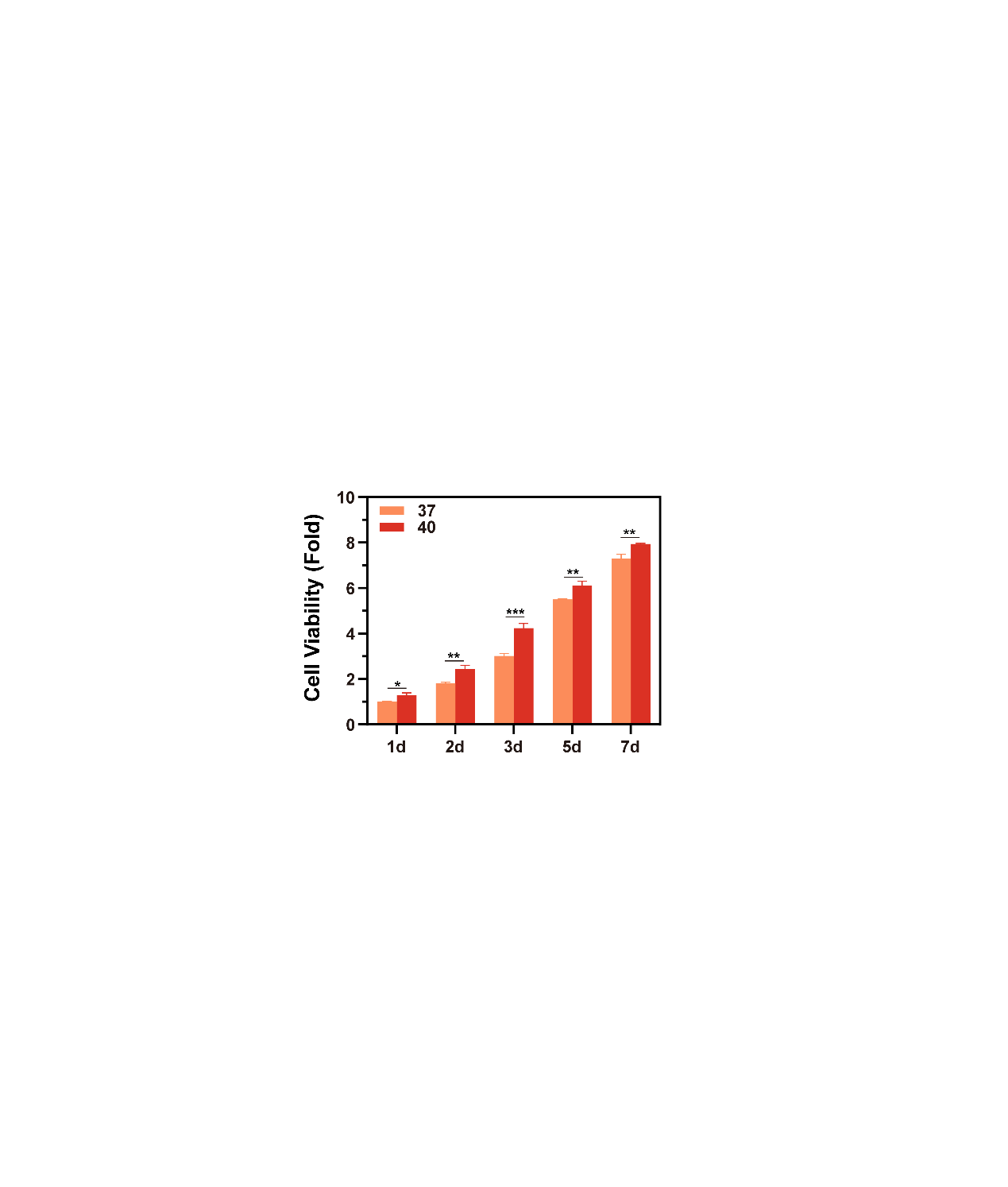


**Figure S17.** Cell proliferation assay of BMSCs under 37 ℃ and 40 ℃ incubation. (Mean ± SD, n=4)


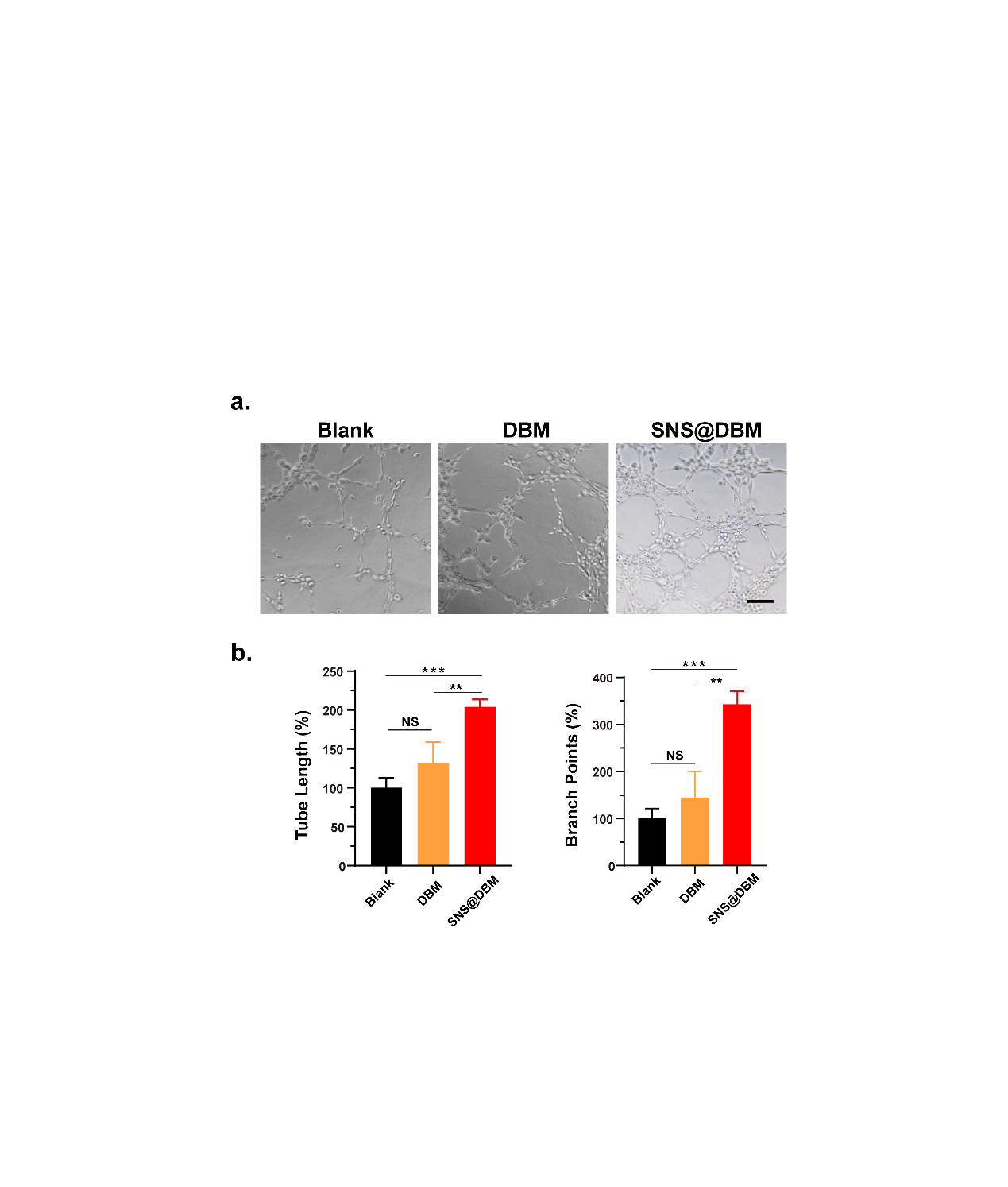


**Figure S18.** The tube formation of HUVEC on matrigel after incubation with ionic extraction for 4 h. a). digital photographs and b). statistical analysis data. Images share the same scale bar of 200 µm. (Mean ± SD, n=3)


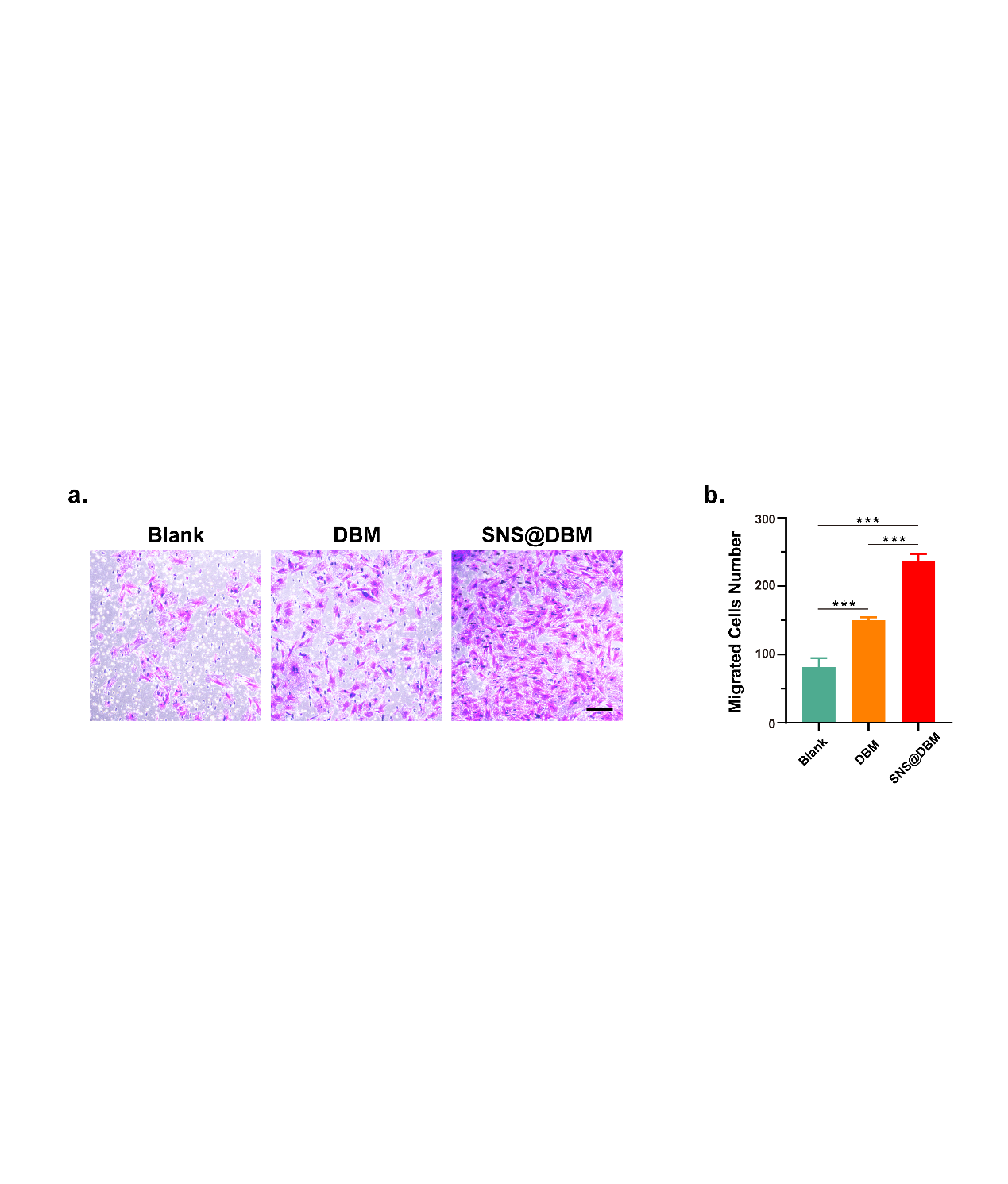


**Figure S19.** The migration number of BMSCs of different treatment groups. a). digital photographs and b). statistical analysis data. Images share the same scale bar of 200 µm. (Mean ± SD, n=3)


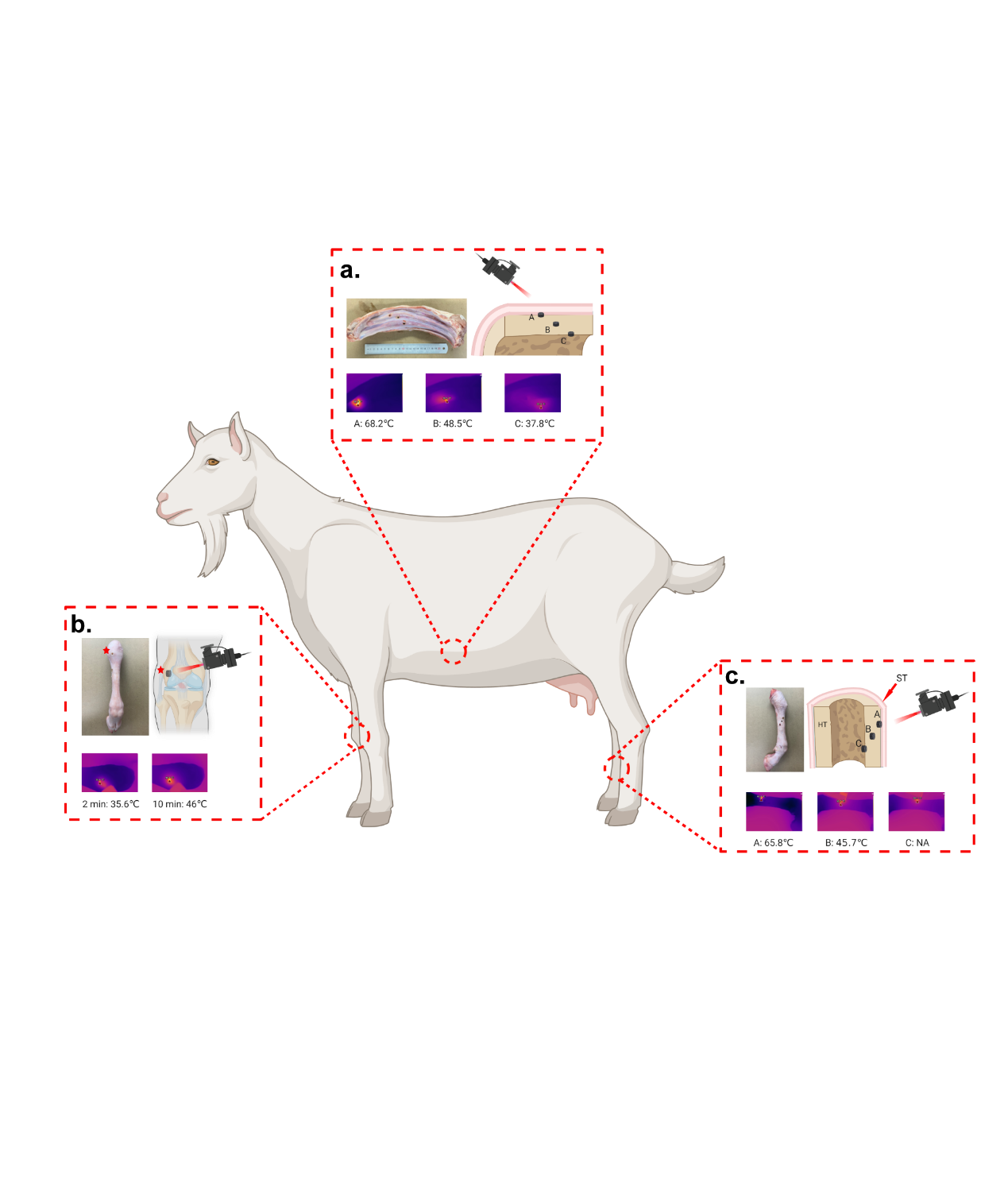


**Figure S20.** Schematic diagram and results of PTT effect on giant animal body models of a). ribs, b). distal femur and c). tibial medullary cavity.

**Table S1.** The binding energy of different docking phases between SNS and COL1A1 chain.

| **Amino acid filed** | **Number of docking phase** | **Energy interval** | **Minimum energy（kJ mol^-1^）** |
| --- | --- | --- | --- |
| TGARGAPGDRGEPGPPGPAGFAGPPGADGQP | 50 | -24.7802428~-23.4826287 | -24.7802428 |
| GADGQPGAKGEPGDTG | 50 | -29.803265 ~ -29.5939724 | -29.803265 |
| PGAKGEPGDTGVKGDAGPPGP | 50 | -28.5893679~27.2080368 | -28.5893679 |
| VKGDAGPPGPAGPAGPPGP | 50 | -27.2080368~-26.9568857 | -27.2080368 |
| PAGPAGPPGPIGNVGAPGPKG | 50 | -27.2080368~-25.8685643 | -27.2080368 |
| IGNVGAPGPKGSRGAAGPPGA | 50 | -26.4545835~-25.7429887 | -26.4545835 |
| GSRGAAGPPGATGFPGAAGRV | 50 | -26.2871494~-24.8639598 | -26.2871494 |
| ATGFPGAAGRVGPPGPSGNAG | 50 | -28.756802~-28.2963583 | -28.756802 |
| VGPPGPSGNAGPPGPPGPVGK | 50 | -26.5383005~-26.0359983 | -26.5383005 |
| GPPGPPGPVGKEGGKGPRGETGPAGRP | 50 | -28.2544998~-27.8777731 | -28.2544998 |

**Table S2.** Primer sequences for genes for qPCR assay

|  | Forward primers (5’-3’) | Reverse primers (5’-3’) |
| --- | --- | --- |
| *OCN* | TCAACAATGGACTTGGAGCCC | GCAACACATGCCCTAAACGG |
| *SPP1* | CCAGCCAAGGACCAACTACA | AGTGTTTGCTGTAATGCGCC |
| *Runx2* | GCGGTGCAAACTTTCTCCAG | TGCAGCCTTAAATGACTCGG |
| *COL1A1* | ATCAGCTGGAGTTTCCGTGC | GGACCCATTGGACCTGAAGC |
| *GAPDH* | CCGCATCTTCTTGTGCAGTG | CGATACGGCCAAATCCGTTC |

Abbreviations are listed in the Appendix.
